# Perinatal risk factors for fecal antibiotic resistance gene patterns in pregnant women and their infants

**DOI:** 10.1371/journal.pone.0234751

**Published:** 2020-06-18

**Authors:** Andrea Sosa-Moreno, Sarah S. Comstock, Kameron Y. Sugino, Teng F. Ma, Nigel Paneth, Yelena Davis, Rosemary Olivero, Rebecca Schein, Joel Maurer, Lixin Zhang

**Affiliations:** 1 Department of Epidemiology and Biostatistics, Michigan State University, East Lansing, MI, United States of America; 2 Department of Food Science and Human Nutrition, Michigan State University, East Lansing, MI, United States of America; 3 Department of Obstetrics, Gynecology, and Reproductive Biology, College of Human Medicine, Michigan State University, East Lansing, MI, United States of America; 4 Helen DeVos Children’s Hospital of Spectrum Health, Grand Rapids, MI, United States of America; 5 Department of Pediatrics and Human Development, College of Human Medicine, Michigan State University, East Lansing, MI, United States of America; Nankai University, CHINA

## Abstract

Perinatal factors can shape fecal microbiome patterns among pregnant women and their infants. However, there is scarce information about the effect of maternal demographics and perinatal exposures on antibiotic resistance genes (ARG) and mobile genetic element (MGE) patterns in pregnant women and infants. We examined fecal samples from pregnant women during their third trimester of pregnancy (n = 51) and 6-month-old infants (n = 40). Of the 91 participants, 72 represented 36 maternal-infant dyads, 15 were additional pregnant women, and 4 were additional infants. We assessed the effects of demographics, pre-pregnancy BMI, smoking and parity in the pregnancy resistome and the effects of demographics, delivery mode, feeding habits and prenatal antibiotic treatment on the infancy resistome. ARG and MGE richness and abundance were assessed using a SmartChip qPCR-array. Alpha diversity (Shannon and Inverse Simpson index) and beta diversity (Sorensen and Bray-Curtis index) were calculated. The Wilcoxon and the Kruskal non-parametric test were used for comparisons. There is a high variability in shared resistome patterns between pregnant women and their infants. An average of 29% of ARG and 24% of MGE were shared within dyads. Infants had significantly greater abundance and higher diversity of ARG and MGE compared to pregnant women. Pregnancy and infancy samples differed in ARG and MGE gene composition and structure. Composition of the fecal resistome was significantly associated with race in pregnant women, with non-white women having different patterns than white women, and, in infants, with extent of solid food consumption. Our data showed that the pregnancy and infancy resistome had different structure and composition patterns, with maternal race and infant solid food consumption as possible contributors to ARG. By characterizing resistome patterns, our results can inform the mechanism of antibiotic resistome development in pregnant women and their infants.

## Introduction

The emergence and spread of antibiotic resistance is a major global public health concern since it presents an obstacle to the treatment and control of infections [1][2]. In developed countries, the use of antibiotics among pregnant women is frequent and has been rising in the past decades, largely to prevent prenatal or postnatal complications such as neonatal Group B *Streptococcus* (GBS) infections, or to reduce the severity of infection after cesarean birth [3][4][5][6]. The rising use of antibiotics has contributed to the epidemic of AMR in the United States [7], and may also cause escalating AMR in pregnant women and their infants.

The presence of antibiotic resistance genes (ARG) in the early gut microbiome of neonates suggests vertical transmission of these genes from mother to child [8][9][10]. Therefore, perinatal exposures may influence the infant resistome, which is defined as the collection of all ARG in the genome of an individual’s microbiome [11]. Mode of delivery and premature birth in early infancy microbiome and resistome acquisition have been reported [5] [10][12][13][14]. Vaginally delivered infants shared more gut microbiome patterns with their mothers than c-section infants, although c-section newborns had higher abundance of ARG than vaginally delivered infants [12]. Preterm 2-day old infants intestinal microbiome showed different patterns compared with full term infants [10]. Maternal gut microbiome and the breast milk microbiome have been proposed as mechanisms for ARG vertical transmission in the postpartum setting [15]. Although ARG have been detected in meconium samples–intestinal contents formed prenatally–*in utero* ARG transmission remains an active research interest [16] [17] [18].

Mobile genetic elements (MGE) are essential for the dispersal of antibiotic resistance throughout the microbiome by transporting genes between members of the gut microbial community [19]. The assortment of MGE in the genome—including transposons, integrons, plasmids and insertion sequences—is known as the mobilome.

Prior research in this area has several limitations. Studies with few measured ARG [8][20][21] or that use culture-dependent techniques [8] preclude a comprehensive understanding of the full microbiome. The use of non-targeted, shotgun metagenomic approaches which may neglect rare DNA sequence targets [12][15][22][23]. A more targeted approach, such as we use, offer a more comprehensive and cost-effective approach to the study of ARG and MGE.

Information is scarce about the resistome patterns in pregnancy and infancy. Furthermore, the impact of maternal demographics and perinatal exposures on ARG and MGE has not been comprehensively assessed. This study provides prospectively collected information about the characteristics of the maternal (third trimester pregnancy) and infancy (6-month old) resistome and mobilome using Takara Smartchip technology. This is a highly sensitive DNA quantification technique, based on a targeted metagenomics approach. An analysis of the effects of demographics as well as pre- and perinatal exposures on the pregnancy and infancy resistome is also provided.

## Materials and methods

### Study population

All samples were collected as part of the ARCH_GUT_ and BABY_GUT_ cohorts in Lansing and Traverse City, MI [24]. The aim of ARCH_GUT_ and BABY_GUT_ cohorts was to understand how maternal and/or child gut microbiome can modify the effect of pregnancy-related weight, weight changes and social circumstances on childhood obesity. Women younger than 18 years, underweight (BMI<18.5) and unable to complete an interview in English, were excluded in both cohorts. Participants provided written consent at enrollment.

**Fig 1:**
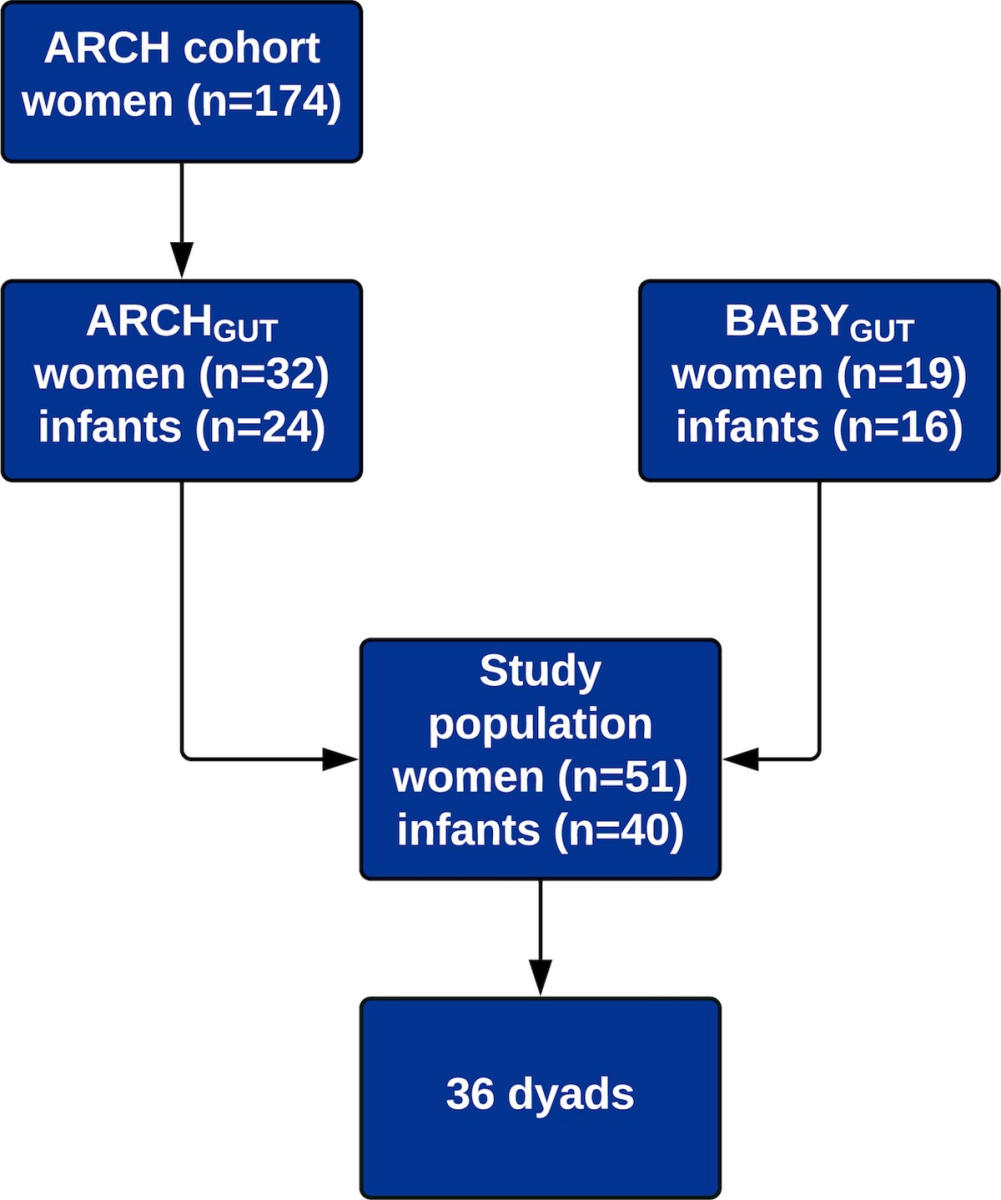
Flowchart of the selection of the study population.

Pregnant women were prospectively followed until they had successful births and the same newborns were followed through 6 months of age. A total of 51 samples from pregnant women and 40 samples from 6-month-old infants, were available for the analysis (Fig 1), included 36 mother-infant dyads. ARCH, ARCH_GUT_ and BABY_GUT_ cohorts were approved by the Michigan State University Institutional Review Board (IRB C07-1201, 15–1240 and 14-170M).

### Antimicrobial resistance genes and mobile genetic elements

Fecal samples from pregnant women were collected during their third trimester of pregnancy. Fecal samples from infants were collected near 6 months of age. MoBio Powersoil DNA Isolation kit (Qiagen MoBio, Carlsbad, CA) was used for DNA extraction of 250mg of fecal sample. More information can be found elsewhere [24]. Takara SmartChip Real-time PCR was used to perform high-parallel quantitative PCR targeting up to 321 ARG and 53 MGE. Bacterial 16S rRNA gene was also included in the Takara SmartChip. Primers for these genes and associated qPCR assay have been designed, used, and validated [25][26][27][28], and recently updated [29]. We randomly selected 12 samples (6 mothers and 6 infants) to be pooled together. After a preliminary run on DNA from those pooled fecal samples, 133 genes (106 ARG and 27 MGE) were present in at least one of the samples and therefore selected for this study (S1 Table). ARG belonged to 8 groups: Aminoglycoside (n = 21), beta-lactamase (n = 16), fluoroquinolone (n = 4), multi-drug resistance (MDR) (n = 21), Macrolide-lincosamide-streptogramin B (MLSB) (n = 18), sulfonamide (n = 5), tetracycline (n = 13) and vancomycin (n = 8) groups. MGE belonged to 4 groups: Transposons related sequences (n = 6), integrase (n = 4), plasmid related sequences (n = 3), insertional sequence (n = 14).

The reaction reagents were prepared using LightCycler^®^ 480 SYBR Green I Master (Roche Diagnostics, Indianapolis, IN) according to the manufacture’s protocol. PCR program used for the SmartChip was: an initial cycle of 2 min 53 sec at 95°C followed by 40 cycles of 95°C for 34 sec and 60°C for 1 min and 4 sec. All qPCR reactions were run in triplicate. To reduce false positive, a Ct of 30 was used as threshold cutoff when assessing absence/presence of genes. The identity of amplicons from this methodology was previously validated by sequencing of amplicons from fecal samples [30].

### Demographics and perinatal covariates

Additional covariates that have been associated with changes in resistome patterns were collected in this study. We assessed maternal self-reported race, age, pre-pregnancy BMI (normal: 18.5≤BMI<25, overweight: 25≤BMI<30, obese: BMI≥30), history of smoking, parity and prenatal antibiotic use using the questionnaire at enrollment. To assess maternal antibiotic exposure, women were asked about the absence/presence of exposure to antibiotics one year prior the date of the questionnaire (before fecal sample collection). Infant’s sex (male/female), mode of delivery (vaginal/C-section), percentage of breastmilk in infants’ diet (<50%/≥50%), infant’s diet (solids included in diet/ solids not included in diet), sample shipping time and antibiotic exposure was assessed using the questionnaire at 6 months after birth. Women were asked about the infant’s medication use at 1 week of birth, at 6 months after birth and 1-month prior the 6-month questionnaire. Infant prenatal antibiotic exposure was assessed using maternal antibiotic exposure, while infants who reported taking antibiotics after birth were classified as postnatal antibiotic exposed.

### Microbiome analysis

Fecal microbiome data of our study population was already available for analysis [24]. For DNA extraction, Sugino et al. used the MoBio Powersoil DNA Isolation kit (Qiagen MoBio, Carlsbad, CA). The V4 hypervariable region of the bacterial 16S rRNA gene was amplified using barcoded primers following the mothur wet lab documentation [31]. Sequences were processed in mothur using the Illumina MiSeq SOP and operational taxonomic unit (OTU) taxonomies were assigned by phylotype using the SILVA reference taxonomy [32]. Further information about microbiome analysis can be found at Sugino, et al. [24].

### Statistical analyses

#### ARG and MGE composition and structure

ARG or MGE gene richness was defined as the total number of ARG/MGE present in each sample. Richness was calculated within each of the eight ARG classes. We used the Wilcoxon rank-sum test (or Mann-Whitney U test) a non-parametric test to test for significant differences between independent groups.

To assess resistome structure, we first calculated the number of genetic copies for each gene based on Real-time PCR Ct values using the following formula:

$$GC=10^{\left((30-CT)/3.3333)\right)}$$ (1)

We calculated relative abundance by normalizing the number of genetic copies using 16S rRNA gene.

$$RA=\frac{{GC}_{ARG\;or\;MGE\;gene}}{{GC}_{16S\;rRNA\;gene}}$$ (2)

Relative abundance was calculated within each of the eight ARG classes.

#### Similarities in ARG and MGE patterns between pregnant and infant resistome

To understand the influence of maternal gut resistome on infants, we compared ARG presence/absence patterns in infants and their pregnant mothers.

Shannon and Inverse Simpson indexes were calculated to assess Alpha-diversity using diversity function from vegan package in R. Sorensen (community composition) and Bray-Curtis (community structure) dissimilarity indexes were calculated to assess Beta-diversity using vegdist function from vegan package. Ordination analysis was performed to study data clustering. We used the cmdscale command in R with Bray-Curtis dissimilarity index to draw Principal Coordinate Analysis (PCoA) graphs. Adonis function was used to test statistically significant differences in Beta diversity by permutational multivariable analysis of variance (Permanova: 9999 permutations). The function adonis2 was used to adjust for covariates in multivariable models. In pregnancy samples we adjusted for maternal age, cohort and shipping time, whereas in infant samples we adjusted for sex, breastfeeding status, delivery mode, cohort and shipping time. We used Benjamini and Hochberg methods for p value correction [33].

Resistome and mobilome Bray-Curtis and Sorensen Beta diversity indexes, using relative abundance and presence/absence data respectively, were calculated between related and unrelated dyads. Diversity matrixes were divided into 1) women and infants from different families, 2) women-women or infant-infant from different families, 3) women and infants from the same family, for all 36 available dyads. Kernel density plots using ggplot2 package in R were performed [15].

#### Correlation matrix between resistome and microbial taxa

To understand if the fecal microbial taxa was responsible for the resistome structure, we performed a Procrustes analysis using the command protest in vegan based on PCoA results from the abundance of OTUs at phyla level and ARG relative abundances. To understand the specifics of the association between resistome and microbial taxa, we identified those OTUs to the genus, family and phylum levels and correlated the bacterial relative abundance at each level with ARG relative abundances using Pearson correlation in R. For this analysis, we chose only those ARG present in at least 50% of the samples to reduce bias due to low frequent ARG. Correlations higher than 0.80 were selected for further exploration using networking approaches. P values lower than 0.05 were considered as statistically significant.

## Results

### Study population characteristics (Fig 1, Table 1)

Fecal samples from 36 dyads, as well as an additional 15 pregnant women and 4 infants who were not part of dyads, were available for this study (total n = 91, Fig 1). Characteristics of the study population are available in Table 1. Most women were white (87%) and had given birth to fewer than 3 infants (80%). More than two-thirds of infants (68%) were male, and 62% were delivered vaginally. Most infants (79%) had been fed solids at the time of sample collection.

**Table 1:** Clinical characteristics from pregnant women and infants with resistome data (n = 91).

**Pregnant women (n = 51)**		
Age, *years*^1^		30.8 (21.6–38.6)
Race^2^		
	White	39/45 (87)
	Non-white	6/45 (13)
Pre-pregnancy BMI^2^		
	Normal or underweight (<25)	18/50 (36)
	Overweight (25 - <30)	13/50 (26)
	Obese (≥30)	19/50 (38)
Smoking status^2^		
	Never	27/49 (55)
Parity^2^		
	1–2	40/50 (80)
	≥3	10/50 (20)
Sample Shipping Time, *days*^1^		4 (0–12)
**Infants (n = 40)**		
Sex^2^		
	Male	27/40 (68)
Delivery mode^2^		
	Vaginal	25/40 (63)
Breastmilk ^2^		
	<50%	15/40 (38)
Diet ^2^		
	No solid food	8/38 (21)
Sample Shipping Time, *days* ^3^		4 (0–22)
Birth weight, *grams* ^1^		3540 (2268–4940)

^1^ median (range)

^2^ n/total n (%)

*Missing data Women: Race (n = 6), BMI (n = 1), Smoking (n = 2), Parity (n = 1), Sample Shipping Time (n = 1).

*Missing data Infants: *Food (n = 2)*, *Birth weight (n = 3)*

### Richness and abundance of ARG and MGE genes among fecal samples

All 133 screened genes were present in at least one of the 51 samples from pregnant women, and 98% of screened genes (131) were found in the 40 infants. Detection in individual women ranged from 11–74 ARG and 1–17 MGE per person. In infants, the respective range of detection was 38–65 ARG and 9–20 MGE per infant. Among the 128 genes present in more than 9 total samples (1% of the 91), ARG median relative abundance was 0.57 (range: 0.05–26.7), while MGE median relative abundance was 0.13 (range: 9x10^-6^–8.6). Tetracycline resistance genes and transposon related sequences were the most abundant (median relative abundance: 0.18 and 0.1, respectively) followed by resistance genes to beta-lactamase (0.07), multiple drug resistance (MDR) (0.06), and macrolide-lincosamide-streptogramin B (MLSB) (0.05). The most prevalent ARG were resistance to MDR (median: 13 genes/sample), followed by MGE (12 genes/sample), aminoglycoside (10 genes/sample), tetracycline (9 genes/sample), MLSB (8 genes/sample) and beta-lactamase (7 genes/sample). Sulfonamide, fluoroquinolone and vancomycin relative abundances were low. TetQ (tetracycline ARG), IS613 (MGE) and tetW (tetracycline ARG) were the three most abundant genes among all samples and were also within the seven most prevalent genes.

### Shared ARG and MGE patterns between pregnant women and their infants (Fig 2)

**Fig 2:**
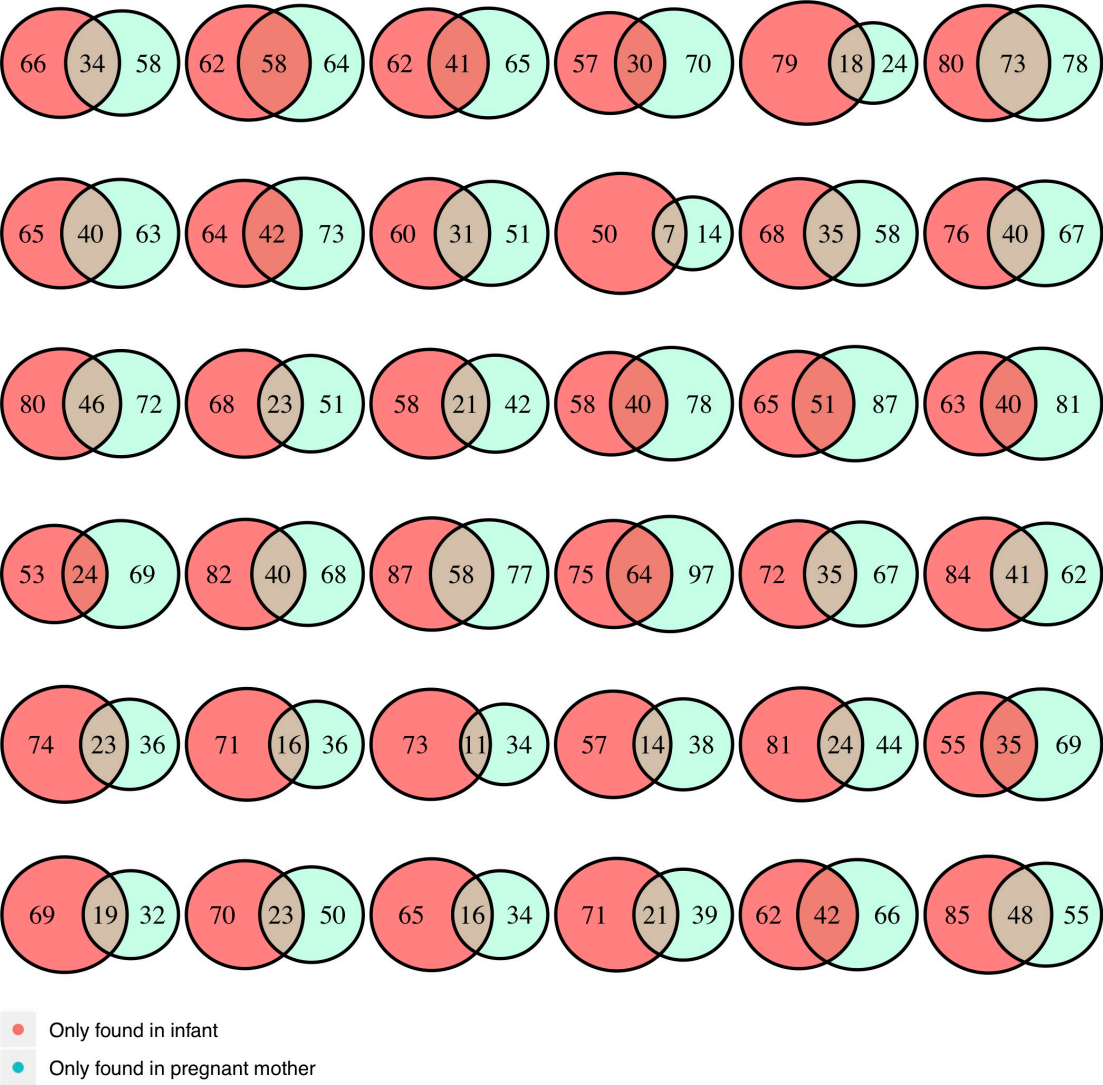
Number of ARG/MGE shared within dyads.

We found a wide range (14% to 94%) of shared genes within dyads, but on average, dyads shared only 29% and 24% of ARG and MGE, respectively (Fig 2). Tetracycline ARG were the most commonly shared genes (50%) within dyads. Two MGE, Intl2 and IncN, were found exclusively in infancy samples, whereas the ARG, aac(6)-im (aminoglucoside), NDM gene (beta-lactamase), and vanTG (vancomycin) were found exclusively in pregnancy samples.

Each circle represents a dyad (n = 36). There is a wide range of shared ARG/MGE patterns between pregnant women and their infants.

### Characteristics of the pregnancy and the infancy resistome (Figs 3, 4A and 4B, S1 Fig)

**Fig 3:**
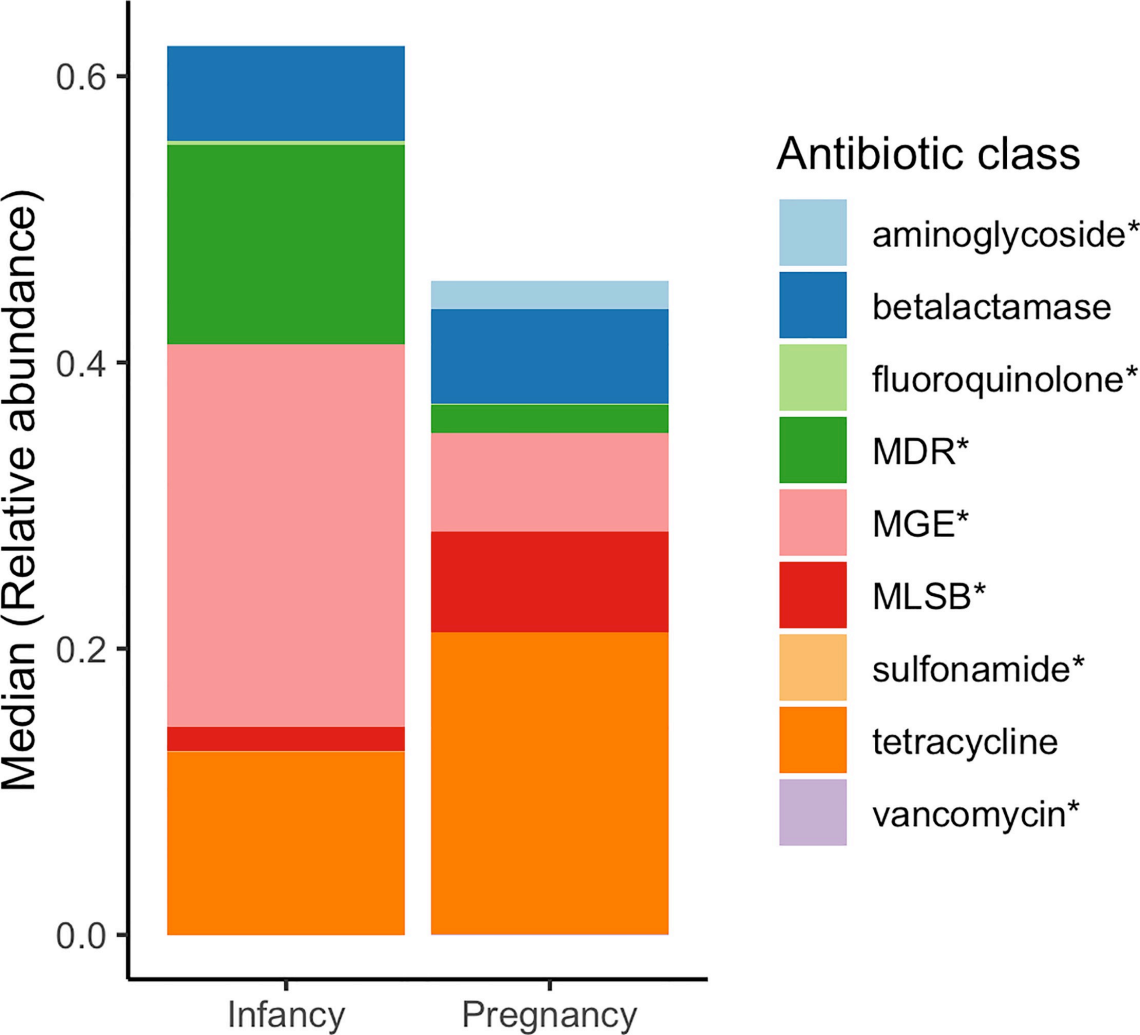
Median relative abundance for each ARG/MGE group in all infant and pregnant samples. The height of the bars represents the median ARG/MGE relative abundance by groups (infant/pregnant). Colors represent different groups of ARG or MGE. Based on all infant (n = 40) and pregnant (n = 51) samples. * represents statistically significant differences between pregnant and infant samples.

**Fig 4:**
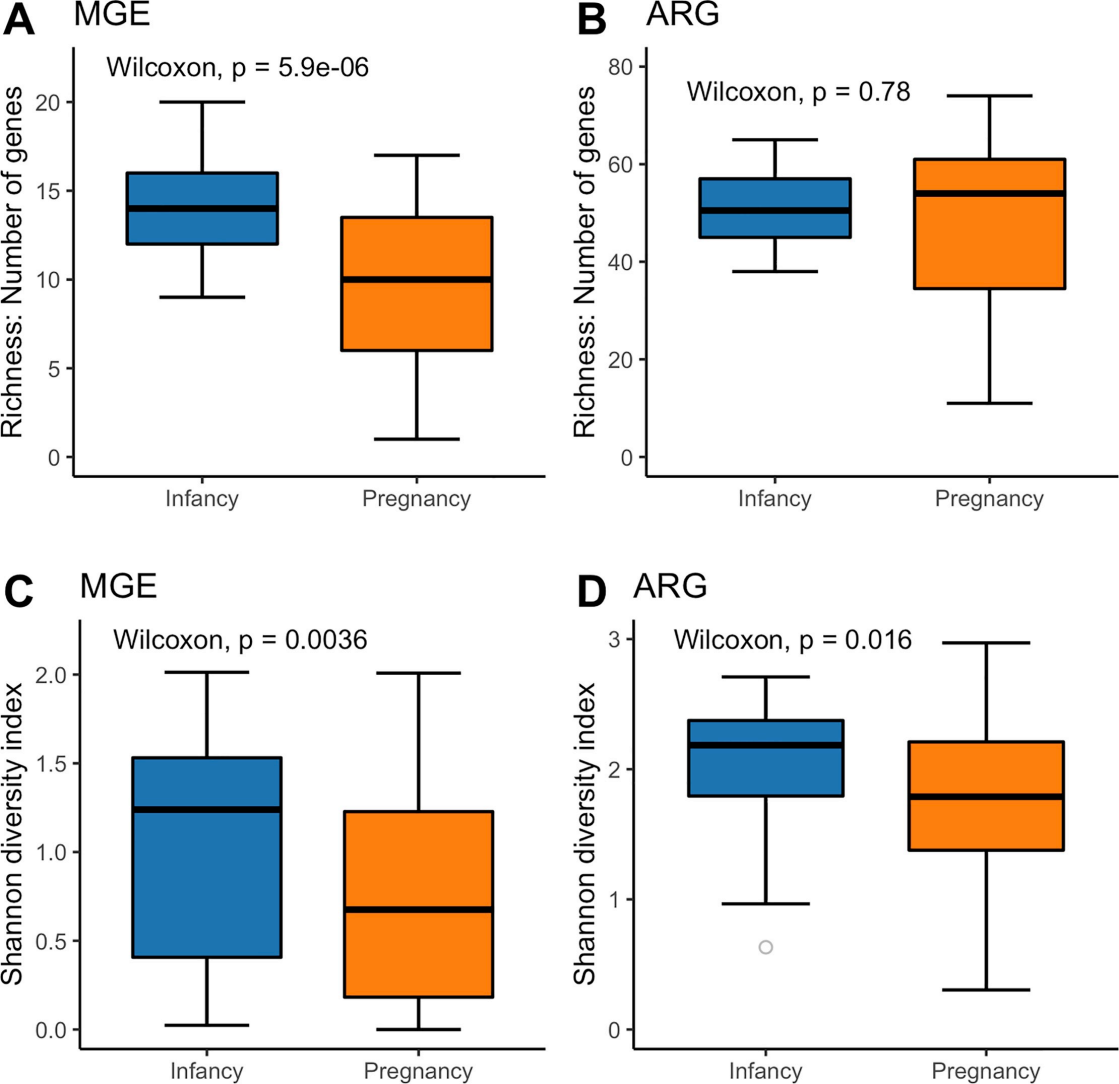
Richness and diversity index comparisons between infants and pregnant women. Based on all infant (n = 40) and pregnant (n = 51) samples. Wilcoxon test was calculated to test for differences between groups.

The sum of the ARG relative abundances, based on all infancy and all pregnancy data (n = 91), was similar in both groups (p = 0.10). However, the pregnancy fecal resistome was significantly enriched with aminoglycoside (p = 1.59x10^-7^), MLSB (p = 9.2x10^-4^) and vancomycin (p = 1.48x10^-9^) ARG compared to the infancy gut resistome. In contrast, MDR (p = 0.008), sulfonamide (p = 2.91x10^-5^), and fluoroquinolone (p = 9.43x10^-5^) were significantly more abundant in the infant resistome (Fig 3). MGE were also more abundant in infants (p = 6.3x10^-4^), this was true for the transposon related sequences (p = 0.013) and for the insertional sequences (p = 2.27x10^-8^). The number of MGE present in infants was higher than in pregnant women, but there was no significant difference in the number of ARG (Fig 4A and 4B). A more detailed view showed that infants had significantly higher number of beta-lactamase ARG (p = 1.5x10^-4^), fluoroquinolone ARG (p = 2.63x10^-5^), MDR (p = 7.5x10^-4^), and sulfonamide ARG (p = 8.1x10^-6^) than did samples from pregnant women. However, pregnant women had significantly higher diversity of aminoglycoside (p = 4.1x10^-6^) and vancomycin (p = 7.9x10^-8^) resistance genes than did infants (S1 Fig). Within the MGE category, infants had a higher number of transposon related genes (p = 1.66x10^-5^) and insertional sequences (p = 1.31x10^-6^) compared with pregnant women.

### Infancy resistome is more diverse than pregnancy resistome: Alpha diversity (Fig 4C and 4D, S2 Fig)

Infants had significantly higher diversity in MGE and ARG relative abundance than pregnant women (Fig 4C and 4D). ARG diversity between pregnant women and infants was also analyzed within the ARG category. Infants had significantly higher Shannon diversity of ARG within the beta-lactamase (p = 0.029), fluoroquinolone (p = 1.78x10^-4^), MDR (p = 0.035), and sulfonamide (p = 3.7x10^-5^) classifications than did samples from pregnant women. However, pregnant women had significantly higher diversity of vancomycin resistance genes than did infants (p = 5.56x10^-7^). These results are consistent with the Inverse Simpson index. There was no difference in gene diversity within aminoglycoside, MLSB or tetracycline resistance genes (S2 Fig).

### Pregnancy resistome differs from infancy resistome: Beta diversity (Fig 5, S3 and S4 Figs)

**Fig 5:**
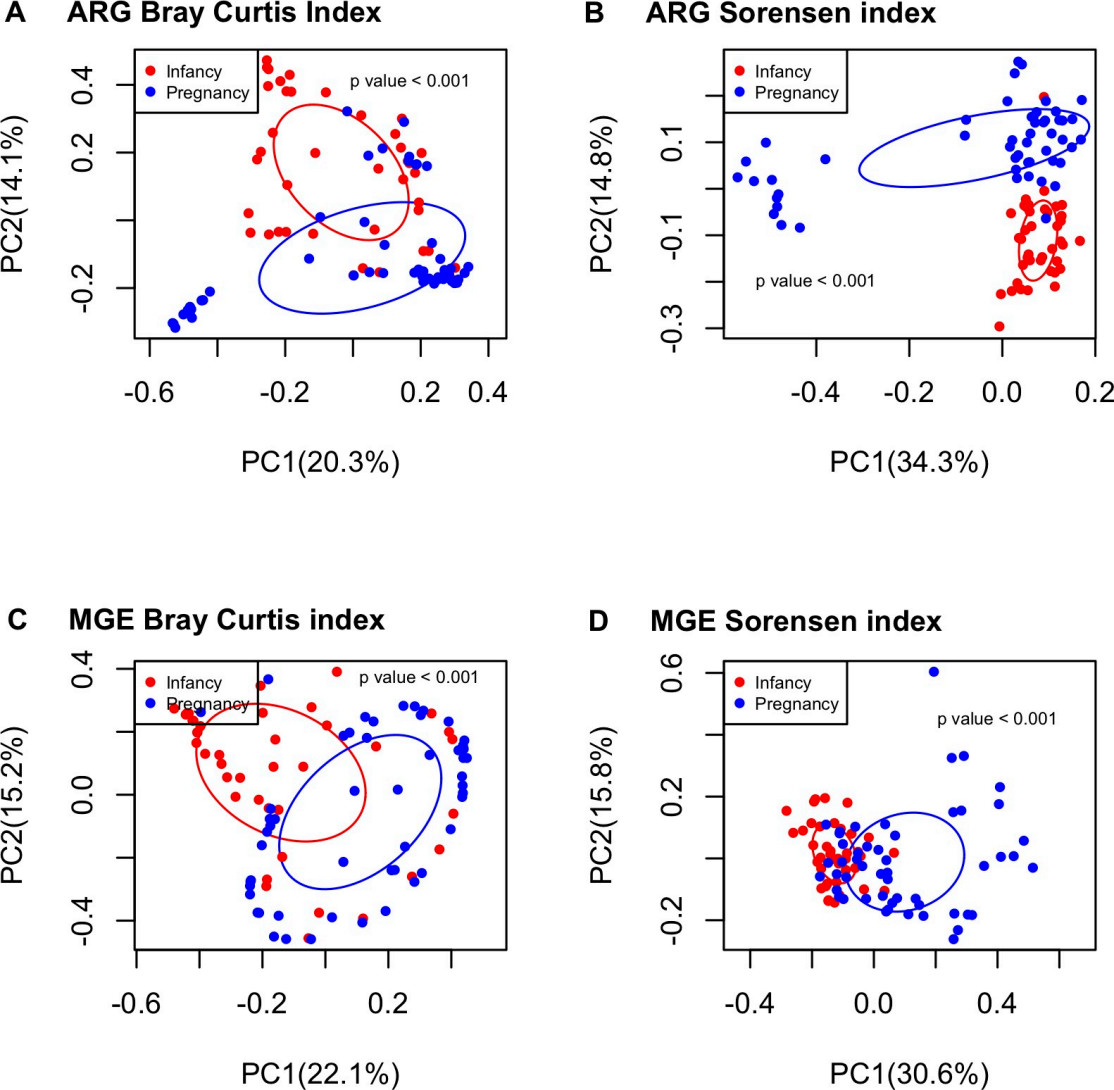
Pregnant samples had different ARG and MGE composition and structure patterns compared to infant samples. PCoA analysis comparing ARG Bray-Curtis (A), ARG Sorensen (B), MGE Bray-Curtis (C) and MGE Sorensen (D) dissimilarity indexes between pregnant (n = 51) and infant (n = 40) samples. Permanova test was calculated to test for differences between groups.

Data were clustered based on Bray-Curtis (structure) and Sorensen (composition) dissimilarity indices. Infancy ARG (Fig 5A) and MGE (Fig 5C) structure patterns were significantly different from pregnancy samples. This clustering was consistent with the composition patterns (Fig 5B and 5D). Both ARG structure and composition ordination plots revealed a distant cluster composed of 12 pregnancy samples whose results did not come from the same experimental run and they did not share a single demographic or other covariate between them. Therefore, the presence of this cluster is not due to any experimental artifacts nor membership in a specific sample group. In both ARG plots, the distant cluster is enriched with erm36 (MLSB ARG), acc3-iva (aminoglycoside ARG), aph6ic (aminoglycoside ARG), aph3-ib (aminoglycoside ARG), vgaB (MLSB ARG), tetG_F genes (tetracycline ARG), among others (S3 Fig).

MGE relative abundance patterns (S4B Fig, family p = 0.03) in infant samples were more similar to those from their own mothers during pregnancy than to those from unrelated pregnant women. Furthermore, infant MGE composition patterns were more similar to those of other infants than to those of their own mothers (S4D Fig, type and family p = 0.014).

### Demographics and perinatal covariates influencing resistome patterns (S2 and S3 Tables, Figs 6 and 7)

**Fig 6:**
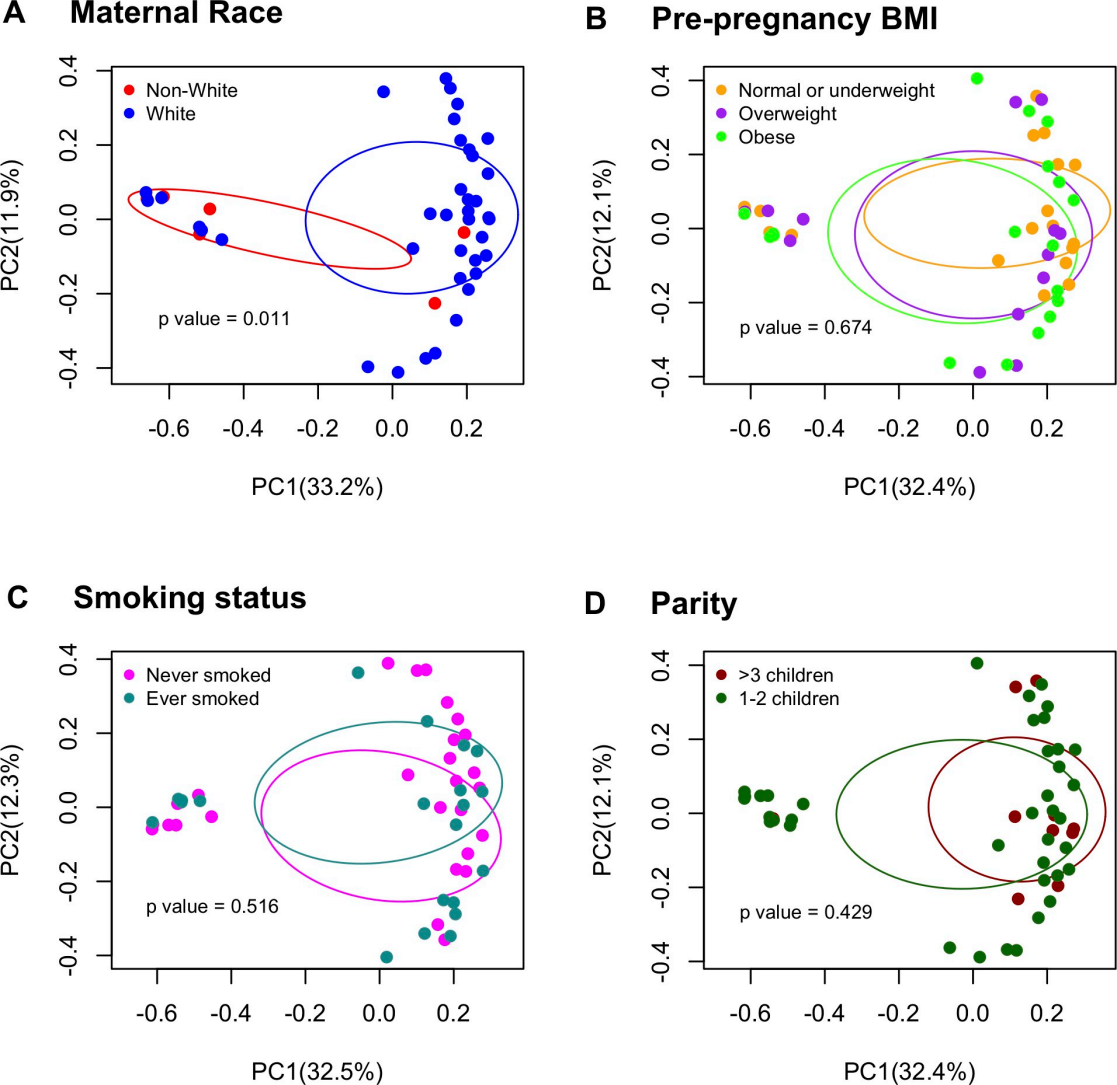
ARG structure by maternal covariates. PCoA plots based on all pregnant (n = 51) samples. Permanova test was calculated to test for differences between groups.

Among pregnancy samples, we found differences in resistome patterns between races. Non-white women had higher ARG abundance (p = 0.062, S2 Table) and lower MGE abundance than white women (p = 0.003, S3 Table). Additionally, white pregnant women had different resistome patterns from non-white women for ARG composition (F = 4.91, p = 0.004) and structure (F = 2.99, p = 0.011) and for MGE composition (F = 2.20, p = 0.027) and structure (F = 2.91, p = 0.013). In contrast, pre-pregnancy BMI, smoking history and parity were not significantly associated with pregnancy ARG/MGE composition and structure patterns (Fig 6). After adjusting for maternal age, pre-pregnancy BMI, cohort and sample shipping time, race was still significantly associated with ARG composition (F = 5.92, p = 0.002), ARG structure (F = 3.35, p = 0.006) and MGE composition (F = 2.85, p = 0.014), but it was not significantly associated with MGE structure (F = 1.79, p = 0.076).

**Fig 7:**
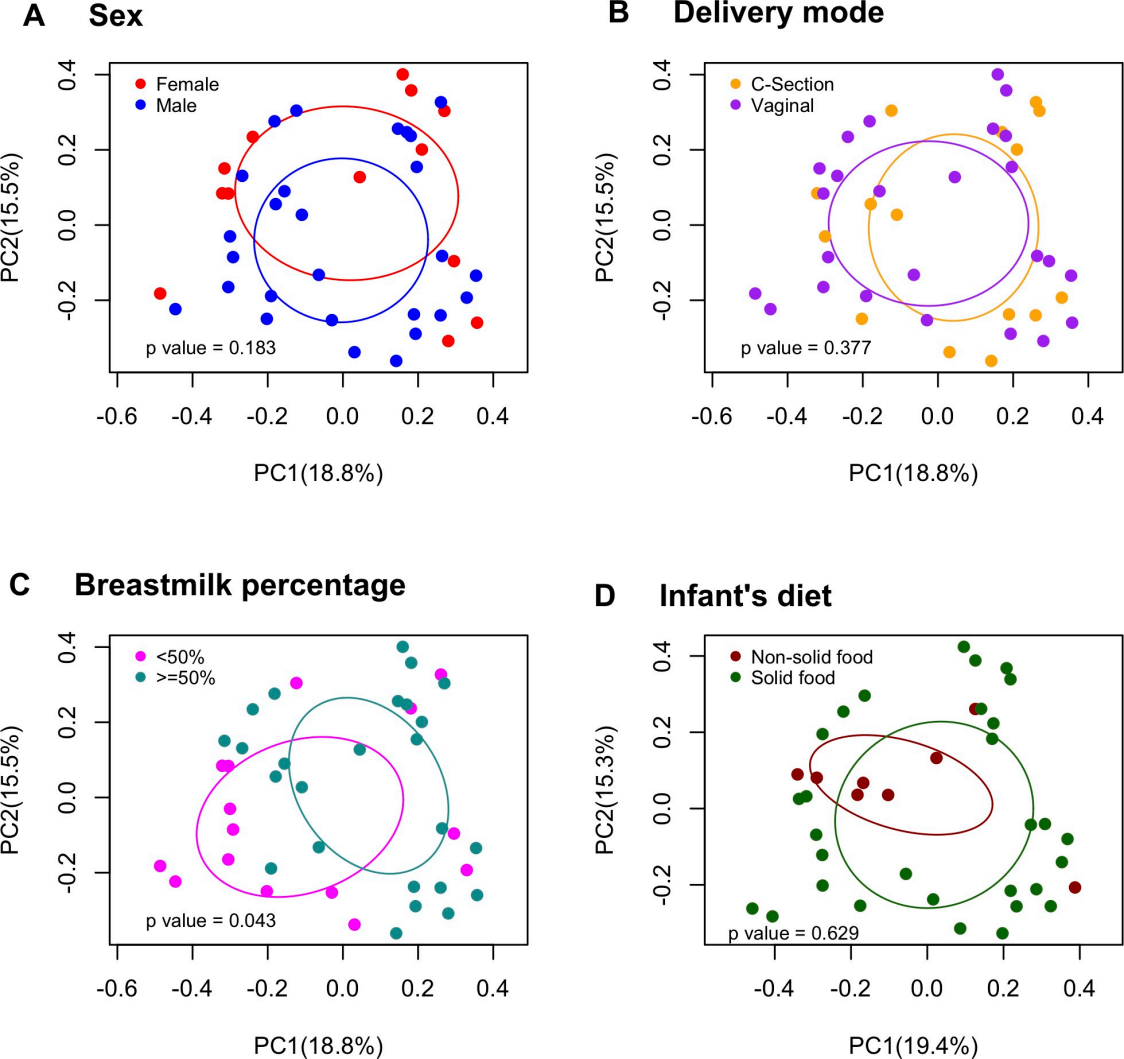
ARG structure by infant covariates. PCoA plots based on all infant (n = 40) samples. Permanova test was calculated to test for differences between groups.

In the univariate analysis of the infancy samples, although not statistically significant, males had higher number of ARG than females (p = 0.056) and infants delivered by c-section had higher number of ARG than vaginally delivered infants (p = 0.059, S2 Table). The ARG structure in infants whose diets were composed of less than 50% breastmilk were different than infants who had diets composed of 50% or more breastmilk (F = 1.75, p = 0.04). MGE composition patterns were different in infants who had added solids into their diets and those who had not (F = 2.24, p = 0.033). Other covariates were not significant in the infancy ARG/MGE composition and structure (Fig 7). After controlling for sex, breastfeeding status, delivery mode, cohort and shipping time, percentage of breastmilk intake was not significant in the multivariable model (F = 1.56, p = 0.08) whereas the presence of solids in the infancy diet showed a borderline significant association (F = 2.05, p = 0.057).

Among 48 women with available antibiotic exposure status, 19 (40%) reported exposure to antibiotics in the year prior to completing the third-trimester questionnaire. We did not find any difference in alpha or beta diversity between pregnant women who reported antibiotic use and those who did not report it. When maternal antibiotic use was matched with their infants, 35 infants had prenatal antibiotic data available, of whom 12 were exposed to antibiotics *in utero*. Six infants reported postnatal antibiotic exposure, 2 of them besides their prenatal exposure. We did not find significant differences in ARG or MGE abundances between infants exposed to antibiotics during pregnancy or after birth and those unexposed.

### Co-occurrence of ARG/MGE in fecal samples (Fig 8A and 8B)

**Fig 8:**
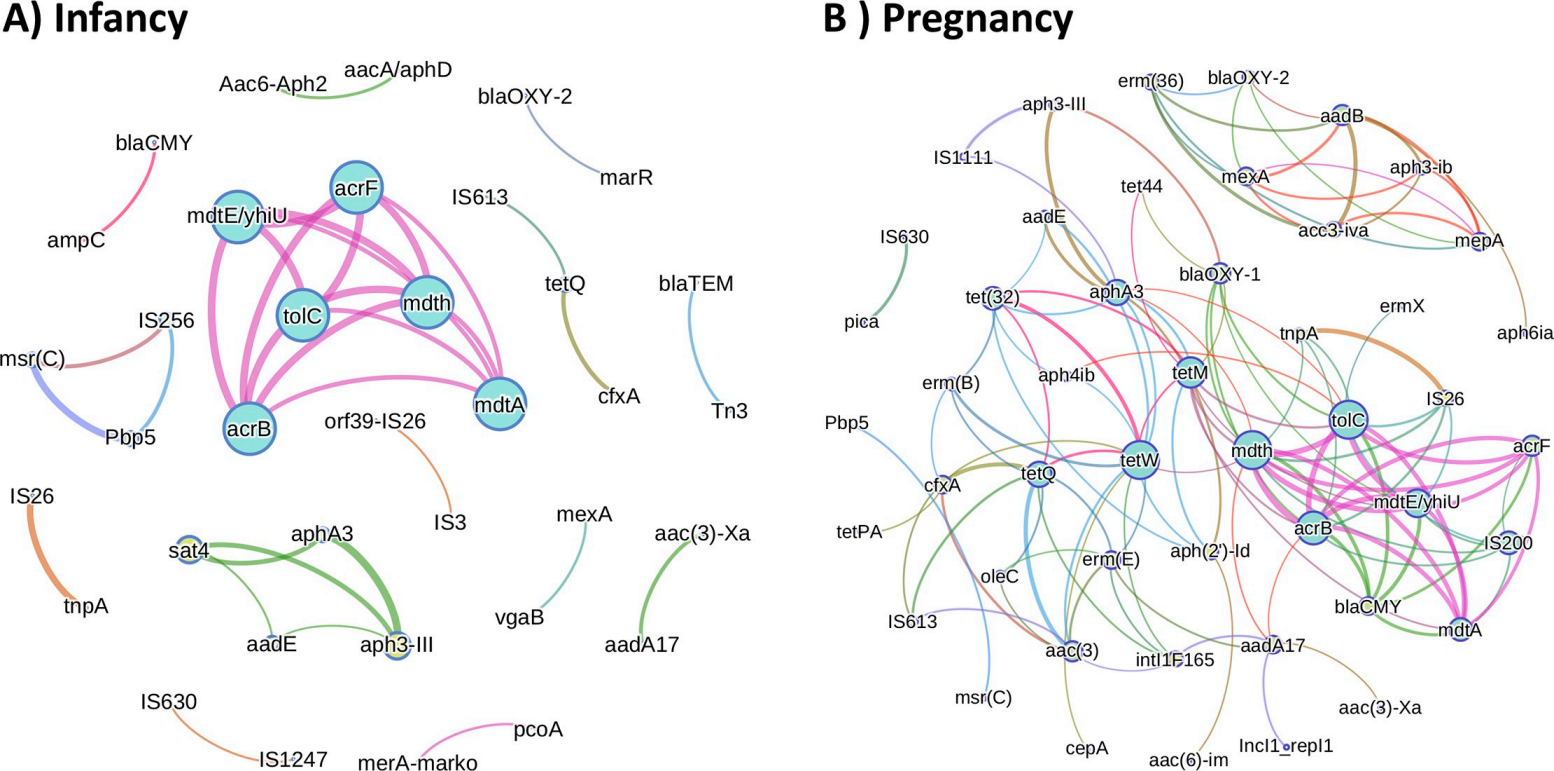
Co-occurrence networking analysis using ARG /MGE log (relative abundances) from infant samples. Nodes (genes) connected by edges represent Spearman correlations higher than 0.6. Node sizes represent degree of centrality (number of connections). The color of the edges represents an antibiotic resistance class and their thickness represent greater correlation coefficients. A) Based on all infancy (n = 40) samples. B) Based on all pregnancy (n = 51) samples.

In networking plots, nodes represent a gene and each edge represent quantitative co-occurrence. We identified two major clusters. The largest cluster was found in both infancy and pregnancy samples and included MDR ARG: tolC, acrB, mdth, acrF, mdtA, mdtE/yhiU (Fig 8A and 8B). The smaller cluster was found only in infant samples and included aminoglycoside ARG: sat4, sphA3, aadE, aph3-III (Fig 8A). Several ARG were correlated with MGE; for instance, pica (MLSB ARG) and IS630, tetW (tetracycline ARG) and intlF165 in pregnancy samples; tetQ (tetracycline ARG) and IS613, msrC (MLSB ARG) and IS256 in infancy samples.

### Gut microbiome is associated with ARG (S4–S9 Tables)

There were 17 different phyla in the infancy and the pregnancy samples. Pregnant women had higher abundance of Firmicutes (p = 2.2x10^-16^), Bacteroides (p = 5.52x10^-12^), Verrucomicrobia (p = 1.88x10^-6^), Synergistetes (p = 7.2x10^-4^), Tenericutes (p = 1.4x10^-3^), Cyanobacteria (p = 1.96x10^-3^), and Lentisphaerae (p = 0.011) phyla compared with infant samples, while infants had higher abundance of Proteobacteria (p = 0.019) and Actinobacteria (8.7x10^-4^) phyla. ARG abundance patterns had a significant association with microbial taxa abundance patterns (Procrustes, p = 0.001) indicating that the microbiome structure is correlated with ARG patterns in fecal samples.

Abundance of several genes were highly correlated with 4 phyla: Fusobacteria, Lentisphaerae, Synergistetes, and Tenericutes in infant and pregnant samples (S4 and S5 Tables). Examples include *Pseudomonas* genera associated with czcA (MDR), and Staphylococcus associated with IS1247 (MGE) and vanHD (vancomycin). Additional correlations higher than 0.8 at the family (S6 and S7 Tables) and genus (S8 and S9 Tables) levels are available in the supplementary information.

## Discussion

Our study describes an exploratory analysis characterizing the resistome of pregnant women and 6-month-old infants. We assessed the effects of age, pre-pregnancy BMI, smoking and parity in the pregnancy resistome and the effects of sex, delivery mode, feeding habits and prenatal antibiotic treatment on the infancy resistome. We found that the fecal resistome of infants had greater alpha and different beta diversity than the pregnancy fecal resistome. In addition, we found that maternal race and solid food consumption may be shaping resistome patterns in pregnant and infant resistome, respectively.

We identified a wide range in shared ARG/MGE between pregnant women and their infants. All infant samples contained some unique ARG compared to those in their own mother’s resistome, suggesting that there are additional sources of ARG at an early age [8]. External factors, such as diet, environment and contact with other persons, could be important in the transmission of ARG from mothers to infants.

Compared to other classes of antibiotics, resistance to tetracyclines and MGE were the most abundant in both the infant and maternal samples. Additionally, beta-lactamase, MDR and MSLB ARG were also abundant. These antibiotics have been prescribed for many decades, so it is understandable that resistance is well-represented in fecal samples [15][23]. Although Tetracycline is contraindicated in pregnancy and in children before age of 8 [34], tetracycline resistance genes have been found in high abundance within fecal samples in infants and pregnant women [8][12][23]. This phenomenon could relate to the wide historical use of tetracycline to treat a variety of infections and to the wide distribution of tetracycline antibiotic resistance genes in various bacterial species [35]. Women could have acquired tetracycline resistance genes before or during their pregnancy from the environment or from their diet, without exposure to the specific antibiotic. Additionally, tetracycline resistance genes constituted the most frequent antibiotic class shared between dyads, suggesting that those genes are prone to vertical transmission explaining their high abundance in infant samples [16]. Vancomycin is commonly used to treat gram positive bacterial infections when other antibiotics are not able to clear the infection [36]. Even though vancomycin ARG have been found in adult samples [37][38], their presence in pregnancy samples is concerning given that vancomycin is sometimes used to treat group B strep infections, an organism screened for in late gestation [39]. There were only 8 vancomycin ARG screened in this study. Moreover, infants had a lower abundance of aminoglycoside ARG compared with pregnant women.

The presence of ARG does not necessarily mean that individuals were exposed to those specific antibiotics, but it could also be the result of horizontal gene transfer within and between bacterial species [37]. Known mechanisms for horizontal gene transfer include MGE, plasmids and prophages [11]. Also, independent acquisition of ARG from food or environmental sources such as the water distribution system [40] or soil [41] cannot be ruled out.

We found that infants had a higher abundance of ARG and MGE than pregnant women. Our results may reflect the difference in microbiome between infants and mothers. The composition of a developing infant gut microbiome is very different from the more stable adult [42]. Those taxa abundant in infants are likely the ones carrying more ARGs and MGEs, explaining our observations. For example, Proteobacteria (Gammaproteobacteria more specifically) are the major carriers for ARGs in studies either employing culture-based [43] or metagenomics method [44]. We did not find any correlation between the phylum Proteobacteria and ARG/MGE, but we found correlations between several bacterial families belonging to the phylum Proteobacteria and ARG/MGE. Additionally, the higher abundance of MGE in infancy samples could facilitate transfer of ARG within and between species [15]. Although our results suggest that the infancy resistome and mobilome are more diverse than those of pregnant women, the literature reports discordant results [15][23].

We found differences between the infant and the adult fecal resistome patterns indicating that the reported divergence between 1 and 2-month-infants and women persists at 6-months [8][15]. Infants MGE relative abundance structure patterns were more similar to the MGE abundance patterns of own mothers than with patterns belonging to unrelated mothers suggesting that either maternal influence and/or family-specific environment could play a role in the acquisition of the mobilome [15]. We also found that infant’s MGE composition patterns were more similar to that of other infants than to that of their own mothers, suggesting that at 6 months-of-age the influence of the environment is more important in the acquisition of the mobilome compared to maternal influence.

Race/ethnicity is associated with human microbiome patterns [45][46][47]. In our study, resistome patterns differ between non-white and white pregnant women. Unfortunately, race/ethnicity encompasses many factors such as socio-economic status, diet, religion, lifestyle and genetic diversity [47] [48] which makes it difficult to specify a reason for our results. Differences may also be due to the small sample size of non-white participants in our study. Further examination of these differences in larger samples is important as the microbiome may be relevant to pregnancy conditions, such as preterm birth, that show marked racial disparities. Regarding infant samples, studies have found a diminishing effect of mode of delivery on the microbiome from birth up to 1-year after delivery [12][49]. We did not find a significant impact of delivery mode on the infant resistome at 6 months.

Antibiotic use creates a selective pressure for drug-resistant strains that are part of the fecal microbiome, promoting antibiotic resistant bacteria colonization and increasing the number and diversity of ARG in antibiotic-exposed groups [50]. This overrepresentation of ARG can persist over time even in the absence of antibiotic-induced selective pressure and therefore it can become a potential threat to the host [51]. Currently, the role of prenatal antibiotic use on the infancy resistome and microbiome is not fully understood [10][15][20][52]. In this study, prenatal antibiotic use was measured as the absence/presence of the exposure by self-report questionnaire. We did not find any relationship between prenatal antibiotic use and resistome patterns.

Co-occurrence analysis between ARG identified two well-defined clusters among infancy samples. The cluster that is characterized by the co-occurrence of ARG sat4, sphA3, aadE and aph3-III has been reported previously by Werner, et al. [53] as part of the transposon Tn5405, from hospital isolates of *Enterococcus faecium*. In addition, the same cluster has been reported to co-occur in fecal samples from broiler chicken slaughterhouses [54].

Fecal microbial taxa were associated with resistome patterns. Significant Procrustes analysis between OTUs and ARG patterns similar to those we detected have been reported [8][37]. Specific ARG were also correlated with some bacterial genera in our data set. In infancy samples, the genus *Escherichia / Shigella* was correlated with 5 MDR ARG: acrF, mdth, mdtE, tolC, and acrB. Previously, Feng et al. found *Escherichia coli* correlated to the presence of 45 ARG using a different panel of genes [37].

The present study has several strengths. We used a culture-independent technique, highly-parallel quantitative real-time PCR, to detect ARG/MGE with high sensitivity. This Smartchip platform allowed us to screen for a total of 133 genes capturing a more representative sample of the resistome. Among our limitations, all ARG and MGE screened via PCR are limited to previously known genes, underestimating the real resistance load within samples. Insufficient data collection regarding GBS prophylaxis, timing, dose and type of antibiotic use may explain why we could not detect differences in ARG/MGE diversity and relative abundances between antibiotic exposed and unexposed groups. Further studies addressing time, dose and type of antibiotic treatment are needed to understand the role of prenatal antibiotic use in the infant resistome, as are larger sample studies to elucidate racial and ethnic differences in the microbiome and their relationship to disparities in perinatal health. Finally, only fecal samples were collected as part of the ARCH_GUT_ and BABY_GUT_ cohorts. Resistome and microbiome analysis of breastmilk, solid food and other environmental samples remain an active research interest [15] [42] [55].

In conclusion, the characterization of resistome patterns can help us understand antibiotic resistome mechanisms in pregnant women and their infants. Our results highlight the differences between the pregnant and infant resistomes and provide information about the associations of specific perinatal factors with the resistome.

## Supporting information

S1 Fig Differences in richness by ARG comparing infant and pregnant samples. Based on all infant (n = 40) and pregnant (n = 51) samples. Wilcoxon test was calculated to test for differences between infant and pregnant samples: ns = not significant p value > 0.05, * = p value < 0.050, ** = p value < 0.01, *** = p value < 0.001, **** = p value < 0,0001.
(TIF)

S2 Fig Differences in Shannon diversity index by ARG comparing infant and pregnant samples. Based on all infant (n = 40) and pregnant (n = 51) samples. Wilcoxon test was calculated to test for differences between infant and pregnant samples: ns = not significant p value > 0.05, * = p value < 0.050, ** = p value < 0.01, *** = p value < 0.001, **** = p value < 0,0001.
(TIFF)

S3 Fig Enrichment of specific ARG/MGE among the distant cluster found in ARG ordination plots. Ordination analysis comparing ARG Bray-Curtis and Sorensen dissimilarity indexes between pregnant (n = 51) and infant (n = 40) samples. Envfit command in R fits ARG in the ordination plot.
(TIF)

S4 Fig Beta diversity dissimilarities between infants and pregnant women resistomes. Based on all infant (n = 40) and pregnant (n = 51) samples. Permanova test was calculated to test for differences between groups. A) Bray Curtis (structure) ARG dissimilarities. B) Bray Curtis (structure) MGE dissimilarities. C) Sorensen (composition) ARG dissimilarities. D) Sorensen (composition) MGE dissimilarities. Type p values represented that infancy and pregnancy resistomes are different. Family p values represented that related dyads are more similar than unrelated dyads. The p value for the interaction between type & family indicated that infant resistomes are more similar to resistomes of other infants than to the resistomes of own mothers.
(TIF)

S1 Table ARG and MGE screened in the Wafergen Smartchip. (PDF)

S2 Table ARG relative abundance, richness, and alpha diversity indexed reported as medians and ranges (Quartile 1-Quartile 3) by demographics and perinatal covariates. (PDF)

S3 Table MGE relative abundance, richness, and alpha diversity reported as medians and ranges of Quartile 1-Quartile 3, by demographics and perinatal covariates. (PDF)

S4 Table Pearson correlation between ARG/MGE and bacteria at phyla level (ρ > 0.8). Infancy samples (n = 41).
(PDF)

S5 Table Pearson correlation between ARG/MGE and bacteria at phyla level (ρ > 0.8). Pregnancy samples (n = 51).
(PDF)

S6 Table Pearson correlation between ARG/MGE and bacteria at family level (ρ > 0.8). Infancy samples (n = 41).
(PDF)

S7 Table Pearson correlation between ARG/MGE and bacteria at family level (ρ > 0.8). Pregnancy samples (n = 51).
(PDF)

S8 Table Pearson correlation between ARG/MGE and bacteria at genus level (ρ > 0.8). Infancy samples (n = 41).
(PDF)

S9 Table Pearson correlation between ARG/MGE and bacteria at genus level (ρ > 0.8). Pregnancy samples (n = 51).
(PDF)

## References

[1] World Health Organization. Antimicrobial resistance: global report on surveillance 2014 [Internet]. 2014. Available from: https://www.who.int/drugresistance/documents/surveillancereport/en/

[2] World Health Organization. Antibiotic resistance [Internet]. 2018. Available from: https://www.who.int/news-room/fact-sheets/detail/antibiotic-resistance

[3] Andrade SE, Gurwitz JH, Davis RL, Chan KA, Finkelstein JA, Fortman K, et al Prescription drug use in pregnancy. Am J Obstet Gynecol. 2004 8;191(2):398–407. 10.1016/j.ajog.2004.04.025 15343213

[4] Broe A, Pottegård A, Lamont R, Jørgensen J, Damkier P. Increasing use of antibiotics in pregnancy during the period 2000–2010: prevalence, timing, category, and demographics. BJOG Int J Obstet Gynaecol. 2014 7;121(8):988–96. 10.1111/1471-0528.12806 24754708

[5] Gasparrini AJ, Crofts TS, Gibson MK, Tarr PI, Warner BB, Dantas G. Antibiotic perturbation of the preterm infant gut microbiome and resistome. Gut Microbes. 2016 9 2;7(5):443–9. 10.1080/19490976.2016.1218584 27472377 PMC5154371

[6] Steinke D, Davey P. Association between Antibiotic Resistance and Community Prescribing: A Critical Review of Bias and Confounding in Published Studies. Clin Infect Dis. 2001 9 15;33(s3):S193–205. 11524719 10.1086/321848

[7] CDC. Antibiotic Resistance Threats in the United States, 2019. [Internet]. Atlanta, GA: U.S. Department of Health and Human Services, CDC; Available from: https://www.cdc.gov/drugresistance/biggest-threats.html?CDC_AA_refVal=https%3A%2F%2Fwww.cdc.gov%2Fdrugresistance%2Fbiggest_threats.html

[8] Moore AM, Ahmadi S, Patel S, Gibson MK, Wang B, Ndao IM, et al Gut resistome development in healthy twin pairs in the first year of life. Microbiome. 2015 12;3(1):27. 26113976 10.1186/s40168-015-0090-9 PMC4480905

[9] Fallani M, Young D, Scott J, Norin E, Amarri S, Adam R, et al Intestinal Microbiota of 6-week-old Infants Across Europe: Geographic Influence Beyond Delivery Mode, Breast-feeding, and Antibiotics: J Pediatr Gastroenterol Nutr. 2010 7;51(1):77–84. 10.1097/MPG.0b013e3181d1b11e 20479681

[10] Arboleya S, Sánchez B, Milani C, Duranti S, Solís G, Fernández N, et al Intestinal Microbiota Development in Preterm Neonates and Effect of Perinatal Antibiotics. J Pediatr. 2015 3;166(3):538–44. 10.1016/j.jpeds.2014.09.041 25444008

[11] Davies J, Davies D. Origins and Evolution of Antibiotic Resistance. Microbiol Mol Biol Rev. 2010 9 1;74(3):417–33. 10.1128/MMBR.00016-10 20805405 PMC2937522

[12] Bäckhed F, Roswall J, Peng Y, Feng Q, Jia H, Kovatcheva-Datchary P, et al Dynamics and Stabilization of the Human Gut Microbiome during the First Year of Life. Cell Host Microbe. 2015 5;17(5):690–703. 10.1016/j.chom.2015.04.004 25974306

[13] Greenwood C, Morrow AL, Lagomarcino AJ, Altaye M, Taft DH, Yu Z, et al Early Empiric Antibiotic Use in Preterm Infants Is Associated with Lower Bacterial Diversity and Higher Relative Abundance of Enterobacter. J Pediatr. 2014 7;165(1):23–9. 10.1016/j.jpeds.2014.01.010 24529620 PMC4074569

[14] Johnson CL, Versalovic J. The Human Microbiome and Its Potential Importance to Pediatrics. PEDIATRICS. 2012 5 1;129(5):950–60. 10.1542/peds.2011-2736 22473366 PMC3340594

[15] Pärnänen K, Karkman A, Hultman J, Lyra C, Bengtsson-Palme J, Larsson DGJ, et al Maternal gut and breast milk microbiota affect infant gut antibiotic resistome and mobile genetic elements. Nat Commun. 2018 12;9(1):3891 10.1038/s41467-018-06393-w 30250208 PMC6155145

[16] Gosalbes MJ, Vallès Y, Jiménez-Hernández N, Balle C, Riva P, Miravet-Verde S, et al High frequencies of antibiotic resistance genes in infants’ meconium and early fecal samples. J Dev Orig Health Dis. 2016 2;7(1):35–44. 10.1017/S2040174415001506 26353938

[17] de Goffau MC, Lager S, Sovio U, Gaccioli F, Cook E, Peacock SJ, et al Human placenta has no microbiome but can contain potential pathogens. Nature. 2019 8;572(7769):329–34. 10.1038/s41586-019-1451-5 31367035 PMC6697540

[18] Kuperman A, Zimmerman A, Hamadia S, Ziv O, Gurevich V, Fichtman B, et al Deep microbial analysis of multiple placentas shows no evidence for a placental microbiome. BJOG Int J Obstet Gynaecol. 2020 1;127(2):159–69. 10.1111/1471-0528.15896 31376240

[19] Ravi A, Avershina E, Foley SL, Ludvigsen J, Storrø O, Øien T, et al The commensal infant gut meta-mobilome as a potential reservoir for persistent multidrug resistance integrons. Sci Rep. 2015 12;5(1):15317. 26507767 10.1038/srep15317 PMC4623605

[20] Gomez-Arango LF, Barrett HL, McIntyre HDavid, Callaway LK, Morrison M, Dekker Nitert M. Antibiotic treatment at delivery shapes the initial oral microbiome in neonates. Sci Rep. 2017 4;7(1):43481. 28240736 10.1038/srep43481 PMC5378909

[21] de Vries LE, Vallès Y, Agersø Y, Vaishampayan PA, García-Montaner A, Kuehl JV, et al The Gut as Reservoir of Antibiotic Resistance: Microbial Diversity of Tetracycline Resistance in Mother and Infant. Gilbert JA, editor. PLoS ONE. 2011 6 28;6(6):e21644 10.1371/journal.pone.0021644 21738748 PMC3125294

[22] Gibson MK, Wang B, Ahmadi S, Burnham C-AD, Tarr PI, Warner BB, et al Developmental dynamics of the preterm infant gut microbiota and antibiotic resistome. Nat Microbiol. 2016 4;1(4):16024. 27572443 10.1038/nmicrobiol.2016.24 PMC5031140

[23] Yassour M, Jason E, Hogstrom LJ, Arthur TD, Tripathi S, Siljander H, et al Strain-Level Analysis of Mother-to-Child Bacterial Transmission during the First Few Months of Life. Cell Host Microbe. 2018 7;24(1):146–154.e4. 10.1016/j.chom.2018.06.007 30001517 PMC6091882

[24] Sugino KY, Paneth N, Comstock SS. Michigan cohorts to determine associations of maternal pre-pregnancy body mass index with pregnancy and infant gastrointestinal microbial communities: Late pregnancy and early infancy. Rosenfeld CS, editor. PLOS ONE. 2019 3 18;14(3):e0213733 10.1371/journal.pone.0213733 30883572 PMC6422265

[25] Guo X, Stedtfeld RD, Hedman H, Eisenberg JNS, Trueba G, Yin D, et al Antibiotic Resistome Associated with Small-Scale Poultry Production in Rural Ecuador. Environ Sci Technol. 2018 8 7;52(15):8165–72. 10.1021/acs.est.8b01667 29944836

[26] Looft T, Johnson TA, Allen HK, Bayles DO, Alt DP, Stedtfeld RD, et al In-feed antibiotic effects on the swine intestinal microbiome. Proc Natl Acad Sci. 2012 1 31;109(5):1691–6. 10.1073/pnas.1120238109 22307632 PMC3277147

[27] Su J-Q, Wei B, Ou-Yang W-Y, Huang F-Y, Zhao Y, Xu H-J, et al Antibiotic Resistome and Its Association with Bacterial Communities during Sewage Sludge Composting. Environ Sci Technol. 2015 6 16;49(12):7356–63. 10.1021/acs.est.5b01012 26018772

[28] Zhu Y-G, Zhao Y, Li B, Huang C-L, Zhang S-Y, Yu S, et al Continental-scale pollution of estuaries with antibiotic resistance genes. Nat Microbiol. 2017 Apr;2(4):16270. 28134918 10.1038/nmicrobiol.2016.270

[29] Stedtfeld RD, Guo X, Stedtfeld TM, Sheng H, Williams MR, Hauschild K, et al Primer set 2.0 for highly parallel qPCR array targeting antibiotic resistance genes and mobile genetic elements. FEMS Microbiol Ecol. 2018 9 1;94(9):fiy130. 10.1093/femsec/fiy130 PMC7250373 30052926

[30] Johnson TA, Stedtfeld RD, Wang Q, Cole JR, Hashsham SA, Looft T, et al Clusters of Antibiotic Resistance Genes Enriched Together Stay Together in Swine Agriculture. mBio. 2016 5 4;7(2):e02214–15, /mbio/7/2/e02214-15.atom. 10.1128/mBio.02214-15 27073098 PMC4959523

[31] Kozich JJ, Westcott SL, Baxter NT, Highlander SK, Schloss PD. Development of a Dual-Index Sequencing Strategy and Curation Pipeline for Analyzing Amplicon Sequence Data on the MiSeq Illumina Sequencing Platform. Appl Environ Microbiol. 2013 9 1;79(17):5112–20. 10.1128/AEM.01043-13 23793624 PMC3753973

[32] Quast C, Pruesse E, Yilmaz P, Gerken J, Schweer T, Yarza P, et al The SILVA ribosomal RNA gene database project: improved data processing and web-based tools. Nucleic Acids Res. 2012 11 27;41(D1):D590–6. 23193283 10.1093/nar/gks1219 PMC3531112

[33] Benjamini Y, Hochberg Y. Controlling the False Discovery Rate: A Practical and Powerful Approach to Multiple Testing. J R Stat Soc Ser B Methodol. 1995;57(1):289–300.

[34] Moreta L, McGovern PG. Antibiotic Use in Pregnancy. Top Obstet Gynecol. 2018 5 15;38(7):1–8.

[35] Karami N, Nowrouzian F, Adlerberth I, Wold AE. Tetracycline Resistance in Escherichia coli and Persistence in the Infantile Colonic Microbiota. Antimicrob Agents Chemother. 2006 1;50(1):156–61. 10.1128/AAC.50.1.156-161.2006 16377681 PMC1346771

[36] Gardete S, Tomasz A. Mechanisms of vancomycin resistance in Staphylococcus aureus. J Clin Invest. 2014 7 1;124(7):2836–40. 10.1172/JCI68834 24983424 PMC4071404

[37] Feng J, Li B, Jiang X, Yang Y, Wells GF, Zhang T, et al Antibiotic resistome in a large-scale healthy human gut microbiota deciphered by metagenomic and network analyses: Antibiotic resistome. Environ Microbiol. 2018 1;20(1):355–68. 10.1111/1462-2920.14009 29194931

[38] Li B, Yang Y, Ma L, Ju F, Guo F, Tiedje JM, et al Metagenomic and network analysis reveal wide distribution and co-occurrence of environmental antibiotic resistance genes. ISME J. 2015 11;9(11):2490–502. 10.1038/ismej.2015.59 25918831 PMC4611512

[39] Hasperhoven G, Al‐Nasiry S, Bekker V, Villamor E, Kramer B. Universal screening versus risk‐based protocols for antibiotic prophylaxis during childbirth to prevent early‐onset Group B streptococcal disease: a systematic review and meta‐analysis. BJOG Int J Obstet Gynaecol. 2020 2 4;1471–0528.16085. 10.1111/1471-0528.16085 PMC7187465 31913562

[40] Xi C, Zhang Y, Marrs CF, Ye W, Simon C, Foxman B, et al Prevalence of Antibiotic Resistance in Drinking Water Treatment and Distribution Systems. Appl Environ Microbiol. 2009 9 1;75(17):5714–8. 10.1128/AEM.00382-09 19581476 PMC2737933

[41] Wang S, Gao X, Gao Y, Li Y, Cao M, Xi Z, et al Tetracycline Resistance Genes Identified from Distinct Soil Environments in China by Functional Metagenomics. Front Microbiol. 2017 7 24;8:1406 10.3389/fmicb.2017.01406 28790997 PMC5522880

[42] Moore RE, Townsend SD. Temporal development of the infant gut microbiome. Open Biol. 2019 9 27;9(9):190128 10.1098/rsob.190128 31506017 PMC6769289

[43] Dantas G, Sommer MOA, Oluwasegun RD, Church GM. Bacteria Subsisting on Antibiotics. Science. 2008 4 4;320(5872):100–3. 10.1126/science.1155157 18388292

[44] Hu Y, Yang X, Li J, Lv N, Liu F, Wu J, et al The Bacterial Mobile Resistome Transfer Network Connecting the Animal and Human Microbiomes. Elkins CA, editor. Appl Environ Microbiol. 2016 11 15;82(22):6672–81. 10.1128/AEM.01802-16 27613679 PMC5086561

[45] Hyman RW, Fukushima M, Jiang H, Fung E, Rand L, Johnson B, et al Diversity of the Vaginal Microbiome Correlates With Preterm Birth. Reprod Sci. 2014 1;21(1):32–40. 10.1177/1933719113488838 23715799 PMC3857766

[46] Hollister EB, Gao C, Versalovic J. Compositional and Functional Features of the Gastrointestinal Microbiome and Their Effects on Human Health. Gastroenterology. 2014 5;146(6):1449–58. 10.1053/j.gastro.2014.01.052 24486050 PMC4181834

[47] Brooks AW, Priya S, Blekhman R, Bordenstein SR. Gut microbiota diversity across ethnicities in the United States. Cadwell K, editor. PLOS Biol. 2018 12 4;16(12):e2006842 10.1371/journal.pbio.2006842 30513082 PMC6279019

[48] for the NutriGen Alliance, Stearns JC, Zulyniak MA, de Souza RJ, Campbell NC, Fontes M, et al Ethnic and diet-related differences in the healthy infant microbiome. Genome Med. 2017 12;9(1):32 10.1186/s13073-017-0421-5 28356137 PMC5372248

[49] Yang R, Gao R, Cui S, Zhong H, Zhang X, Chen Y, et al Dynamic signatures of gut microbiota and influences of delivery and feeding modes during the first 6 months of life. Physiol Genomics. 2019 8 1;51(8):368–78. 10.1152/physiolgenomics.00026.2019 31226006

[50] Oldenburg CE, Hinterwirth A, Sié A, Coulibaly B, Ouermi L, Dah C, et al Gut resistome after oral antibiotics in preschool children in Burkina Faso: A randomized controlled trial. Clin Infect Dis. 2019 5 31;ciz455. 10.1093/cid/ciz455 PMC7456340 31149703

[51] Jernberg C, Löfmark S, Edlund C, Jansson JK. Long-term impacts of antibiotic exposure on the human intestinal microbiota. Microbiology. 2010 11 1;156(11):3216–23. 20705661 10.1099/mic.0.040618-0

[52] Rose G, Shaw AG, Sim K, Wooldridge DJ, Li M-S, Gharbia S, et al Antibiotic resistance potential of the healthy preterm infant gut microbiome. PeerJ. 2017 1 25;5:e2928 10.7717/peerj.2928 28149696 PMC5270596

[53] Werner G, Hildebrandt B, Witte W. Aminoglycoside-Streptothricin Resistance Gene Cluster aadE-sat4-aphA-3 Disseminated among Multiresistant Isolates of Enterococcus faecium. Antimicrob Agents Chemother. 2001 11 1;45(11):3267–9. 10.1128/AAC.45.11.3267-3269.2001 11600397 PMC90823

[54] Qin S, Wang Y, Zhang Q, Chen X, Shen Z, Deng F, et al Identification of a Novel Genomic Island Conferring Resistance to Multiple Aminoglycoside Antibiotics in Campylobacter coli. Antimicrob Agents Chemother. 2012 10;56(10):5332–9. 10.1128/AAC.00809-12 22869568 PMC3457361

[55] Laursen MF, Bahl MI, Michaelsen KF, Licht TR. First Foods and Gut Microbes. Front Microbiol [Internet]. 2017 3 6 [cited 2020 Apr 22];8 Available from: http://journal.frontiersin.org/article/10.3389/fmicb.2017.00356/full 28321211 10.3389/fmicb.2017.00356 PMC5337510

