# Supplementary figures and images for: Perinatal risk factors for fecal antibiotic resistance gene patterns in pregnant women and their infants
Source: PLoS One. 2020 Jun 18;15(6):e0234751. doi: 10.1371/journal.pone.0234751 (PMC7302573; doi:10.1371/journal.pone.0234751)

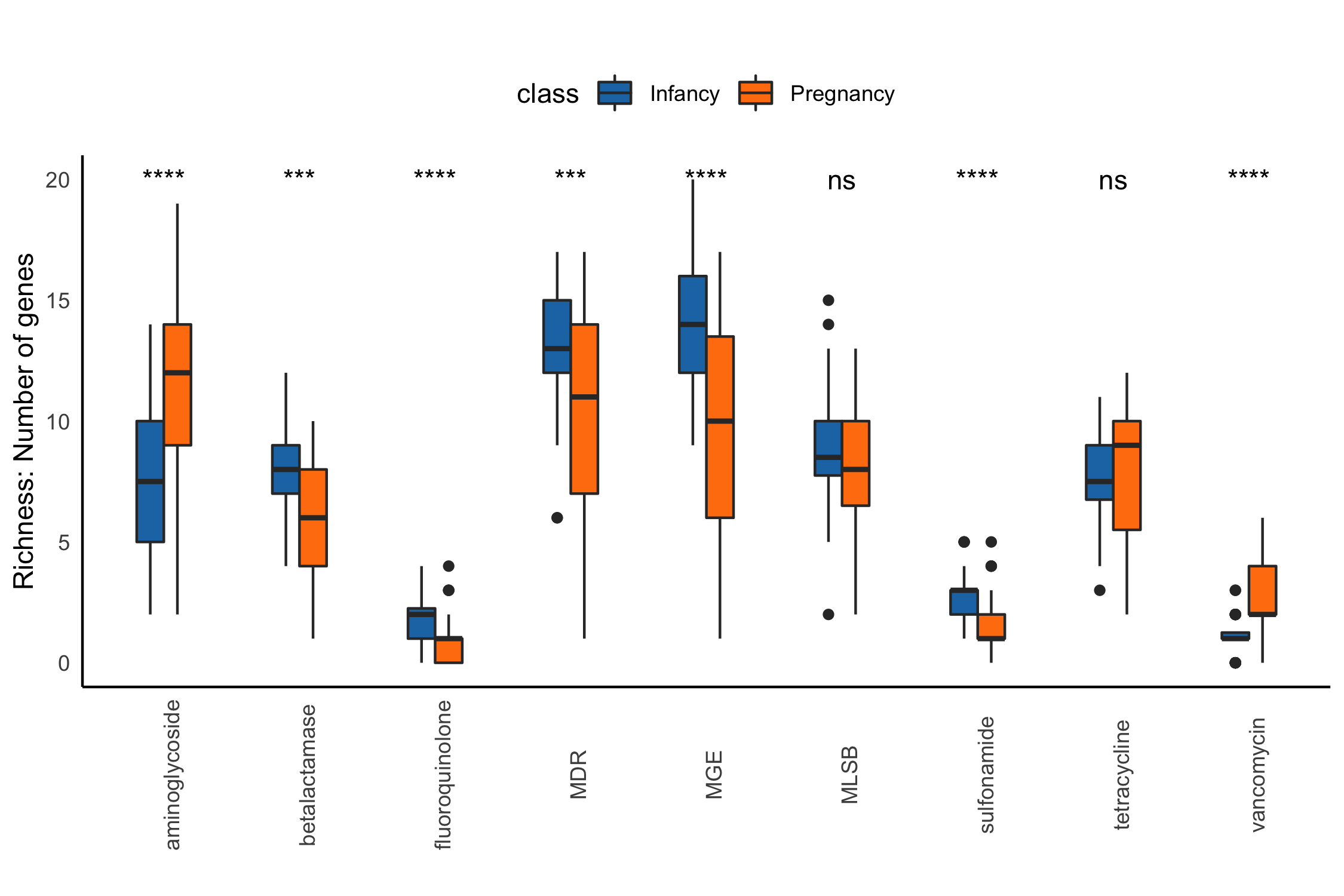

Supplement: S1 Fig — Based on all infant (n = 40) and pregnant (n = 51) samples. Wilcoxon test was calculated to test for differences between infant and pregnant samples: ns = not significant p value > 0.05, * = p value < 0.050, ** = p value < 0.01, *** = p value < 0.001, **** = p value < 0,0001. (TIF) [file pone.0234751.s001.tif]

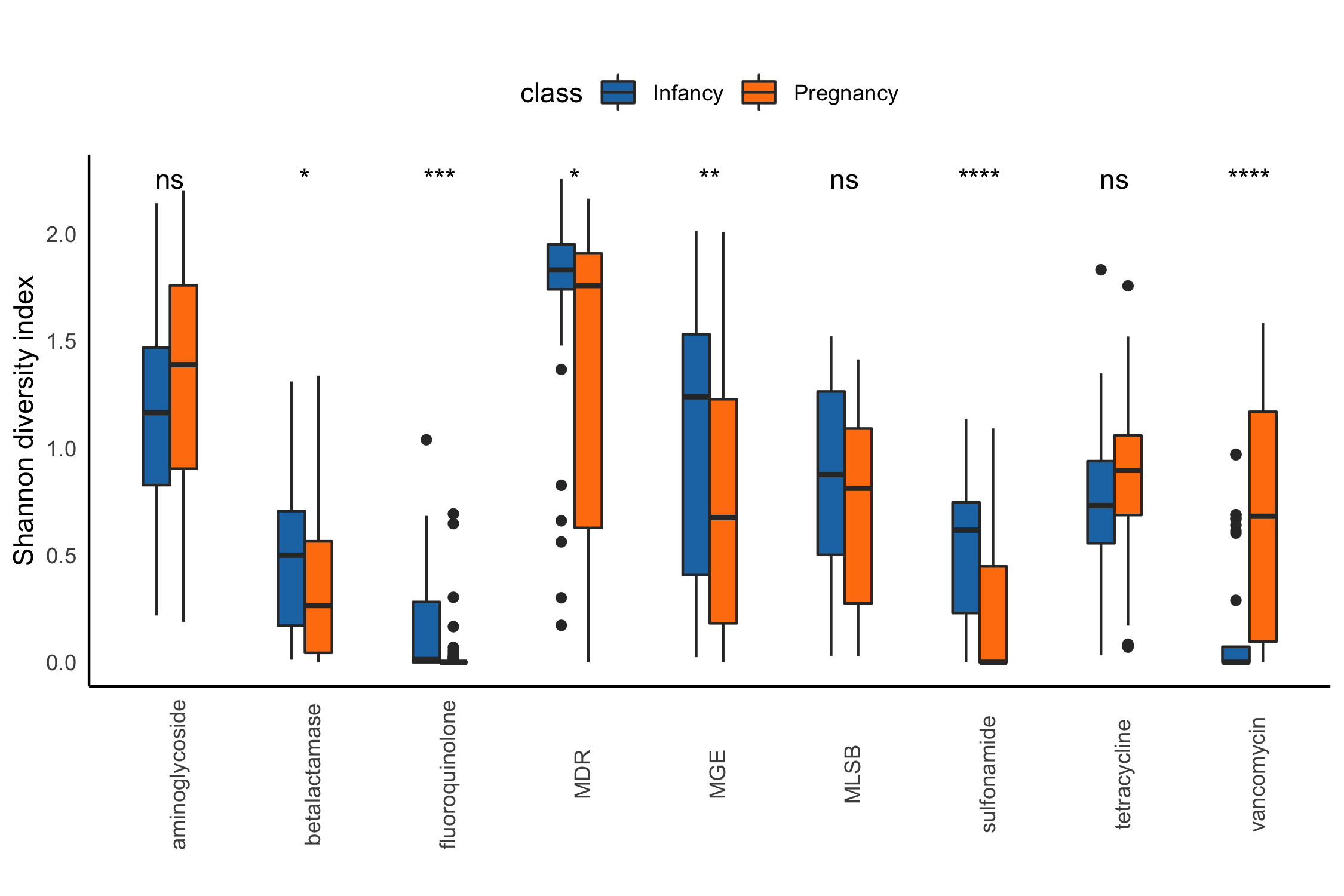

Supplement: S2 Fig — Based on all infant (n = 40) and pregnant (n = 51) samples. Wilcoxon test was calculated to test for differences between infant and pregnant samples: ns = not significant p value > 0.05, * = p value < 0.050, ** = p value < 0.01, *** = p value < 0.001, **** = p value < 0,0001. (TIFF) [file pone.0234751.s002.tiff]

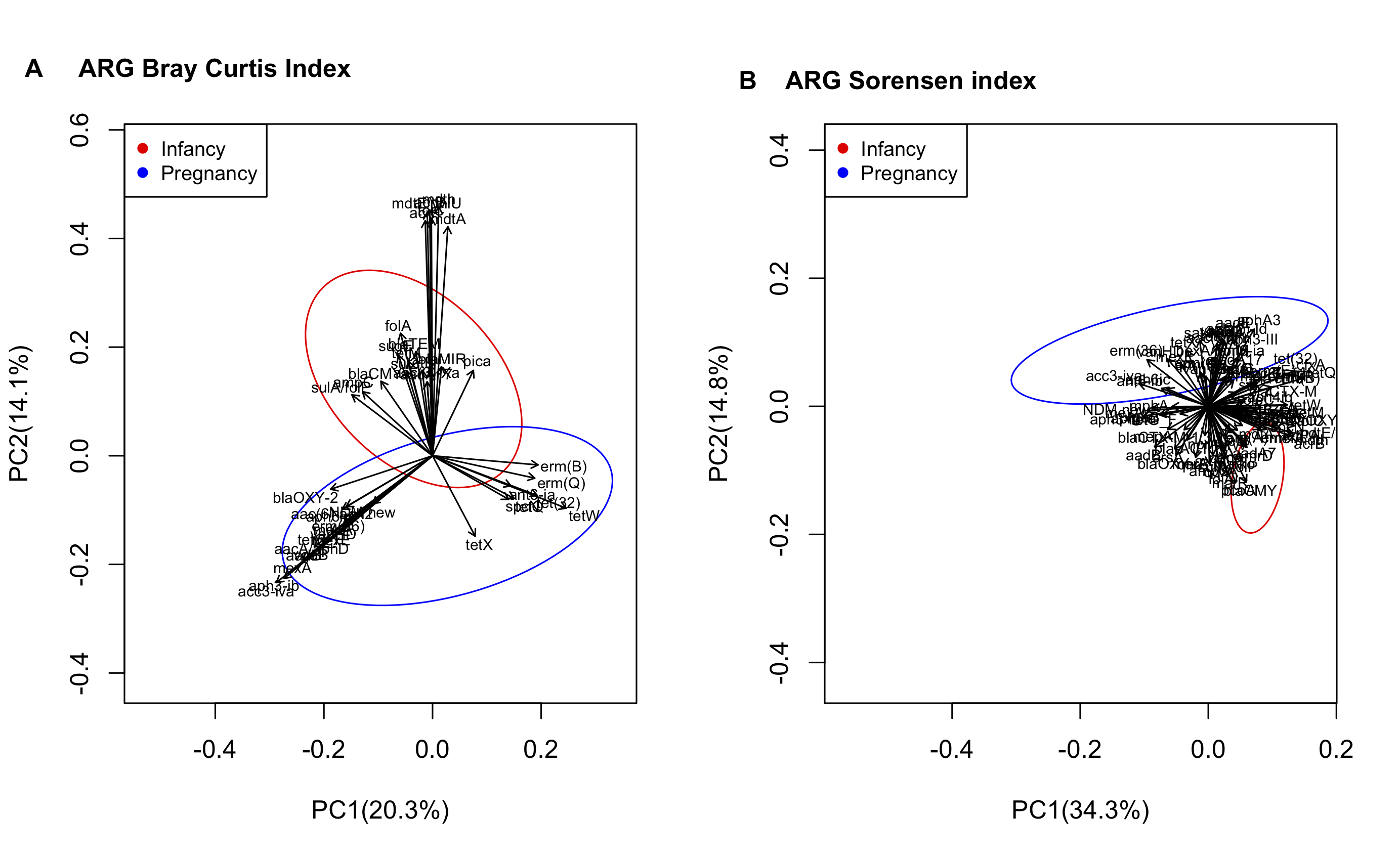

Supplement: S3 Fig — Ordination analysis comparing ARG Bray-Curtis and Sorensen dissimilarity indexes between pregnant (n = 51) and infant (n = 40) samples. Envfit command in R fits ARG in the ordination plot. (TIF) [file pone.0234751.s003.tif]

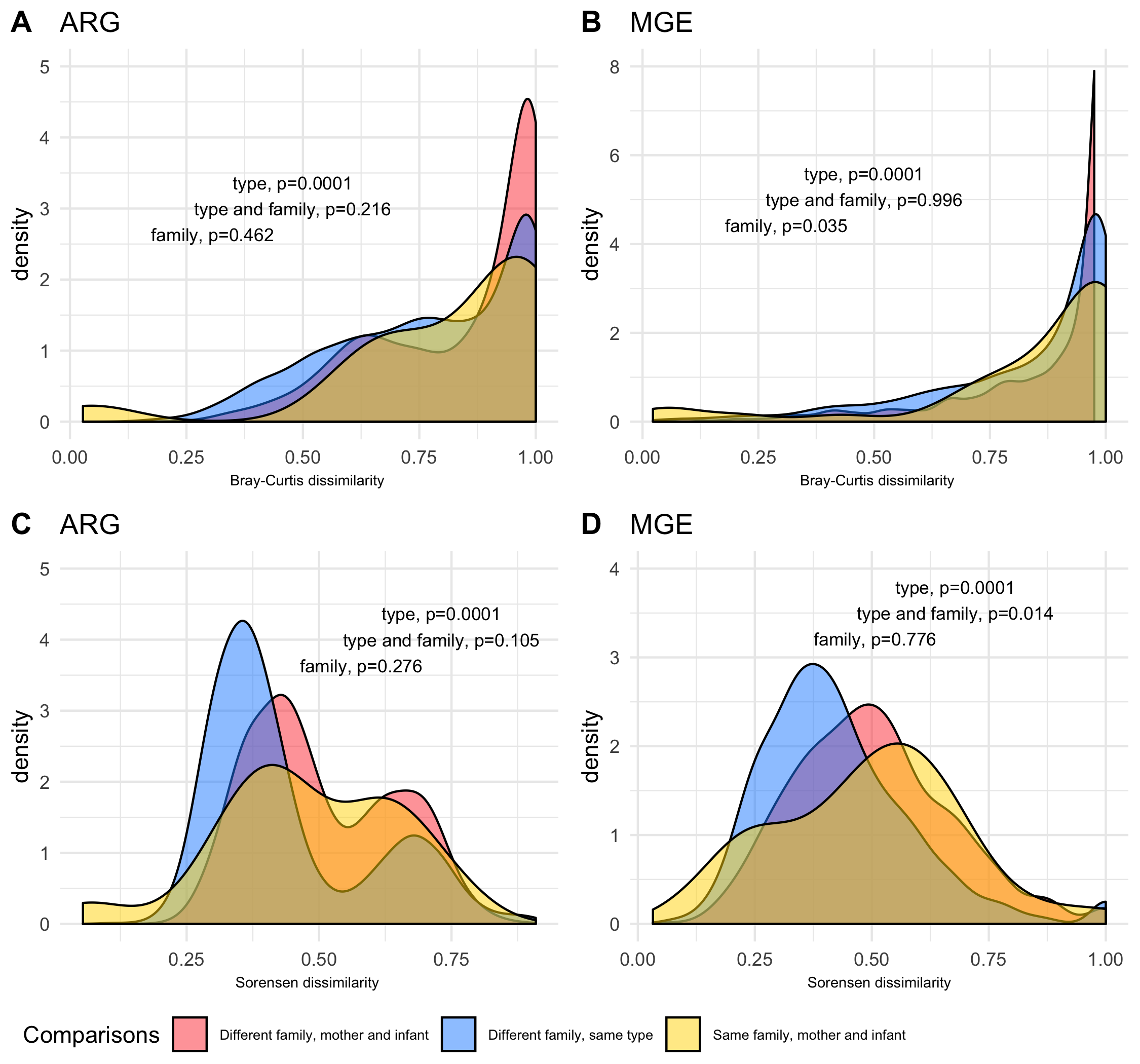

Supplement: S4 Fig — Based on all infant (n = 40) and pregnant (n = 51) samples. Permanova test was calculated to test for differences between groups. A) Bray Curtis (structure) ARG dissimilarities. B) Bray Curtis (structure) MGE dissimilarities. C) Sorensen (composition) ARG dissimilarities. D) Sorensen (composition) MGE dissimilarities. Type p values represented that infancy and pregnancy resistomes are different. Family p values represented that related dyads are more similar than unrelated dyads. The p value for the interaction between type & family indicated that infant resistomes are more similar to resistomes of other infants than to the resistomes of own mothers. (TIF) [file pone.0234751.s004.tif]
