# Supplementary material for: Perinatal risk factors for fecal antibiotic resistance gene patterns in pregnant women and their infants
Source: PLoS One. 2020 Jun 18;15(6):e0234751. doi: 10.1371/journal.pone.0234751 (PMC7302573; doi:10.1371/journal.pone.0234751)
Supplement: S1 Table — (PDF) [file pone.0234751.s005.pdf]

N °	Name	Functional classification	Target antibiotics
1	aacA/aphD	deactivate	Aminoglycoside
2	aphA3	deactivate	Aminoglycoside
3	sat4	deactivate	Aminoglycoside
4	aph(2')-Id	deactivate	Aminoglycoside
5	aadE	deactivate	Aminoglycoside
6	aph4ib	aph4ib	Aminoglycoside
7	aph6ic	aph6ic	Aminoglycoside
8	spcN	deactivate	Aminoglycoside
9	aac(3)	aac(3)	Aminoglycoside
10	Aac6-Aph2	Aac6-Aph2	Aminoglycoside
11	aac(6)-im	aac(6)-im	Aminoglycoside
12	aadA7	aadA7	Aminoglycoside
13	aadA17	aadA17	Aminoglycoside
14	aadB	aadB	Aminoglycoside
15	ant6-ia	ant6-ia	Aminoglycoside
16	aph3-ib	aph3-ib	Aminoglycoside
17	acc3-iva	deactivate	Aminoglycoside
18	aph6ia	deactivate	Aminoglycoside
19	aph3-III	aph3-III	Aminoglycoside
20	ant6-ib	aph3-III	Aminoglycoside
21	aac(3)-Xa	aac(3)-Xa	Aminoglycoside
22	blaOXY-2	deactivate	Beta-lactamase
23	cphA	deactivate	Beta-lactamase
24	cfxA	deactivate	Beta-lactamase
25	cepA	deactivate	Beta-lactamase
26	blaCMY	deactivate	Beta-lactamase
27	blaSFO	deactivate	Beta-lactamase
28	Pbp5	protection	Beta-lactamase
29	blaCTX-M	deactivate	Beta-lactamase
30	penA	protection	Beta-lactamase
31	NDM new	deactivate	Beta-lactamase
32	blaCTX-M-1,3,15	blaCTX-M-1,3,15	Beta-lactamase
33	blaOXY-1	deactivate	Beta-lactamase
34	blaMIR	bla_MIR	Beta-lactamase
35	ampC	deactivate	Beta-lactamase
36	blaTEM	deactivate	Beta-lactamase

37	bla-ACT	bla-ACT	Beta-lactamase
38	qnrB-bob_redesign	efflux	Fluoroquinolone
39	norA	norA	Fluoroquinolone
40	qepA_1_2	qepA_1_2	Fluoroquinolone
41	oqxA		Fluoroquinolone
42	acrB	efflux	MDR
43	acrF	efflux	MDR
44	emrD	efflux	MDR
45	mdtE/yhiU	efflux	MDR
46	mexA	efflux	MDR
47	oprD	efflux	MDR
48	mepA	efflux	MDR
49	mexE	efflux	MDR
50	tolC	efflux	MDR
51	merA-marko	unknown	MDR
52	marR	regulator	MDR
53	mdth	mdth	MDR
54	mdtg	mdtg	MDR
55	pcoA	pcoA	MDR
56	arsA	arsA	MDR
57	bexA/norM	efflux	MDR
58	mdtA	efflux	MDR
59	czcA	czcA	MDR
60	sugE	sugE	MDR
61	terW		MDR
62	pbrT		MDR
63	IS613	MGE	MGE
64	tnpA	MGE	MGE
65	tnpA	MGE	MGE
66	tnpA	MGE	MGE
67	int1-a-marko	MGE	MGE
68	intl2	MGE	MGE
69	IncN_rep	MGE	MGE
70	IncP_oriT	MGE	MGE
71	intI1F165_clinical	MGE	MGE
72	orf39-IS26	MGE	MGE
73	ISSm2-Xanthob	MGE	MGE
74	ISEfm1-Entero	MGE	MGE
75	IS1111	MGE	MGE

76	intl3	MGE	MGE
77	ISCR1		MGE
78	IS26	IS26	MGE
79	IS3	IS3	MGE
80	IS256	IS256	MGE
81	IS200_1	IS200_1	MGE
82	IS1247	IS1247	MGE
83	IS630	IS630	MGE
84	TN5403		MGE
85	IS200		MGE
86	IS21-ISAs29		MGE
87	Tn3		MGE
88	IncI1_repI1		MGE
89	IS91		MGE
90	erm(36)	protection	MLSB
91	ermT	protection	MLSB
92	msr(C)	msr(C)	MLSB
93	ermX	protection	MLSB
94	vgaB	efflux	MLSB
95	pica	protection	MLSB
96	ermA/ermTR	protection	MLSB
97	oleC	efflux	MLSB
98	ere(A)	ere(A)	MLSB
99	erm(B)	erm(B)	MLSB
100	erm(E)	erm(E)	MLSB
101	erm(Q)	erm(Q)	MLSB
102	mphA	deactivate	MLSB
103	erm(35)	erm(35)	MLSB
104	erm(F)	erm(F)	MLSB
105	lsa(C)	lsa(C)	MLSB
106	mefA	efflux	MLSB
107	lnuC	deactivate	MLSB
108	sul2	protection	Sulfonamide
109	strB	protection	Sulfonamide
110	folA	protection	Sulfonamide
111	sulA/folP	protection	Sulfonamide
112	sul1 NEW	protection	Sulfonamide
113	tet(32)	protection	Tetracycline
114	tetA	efflux	Tetracycline
115	tetB	efflux	Tetracycline

116	tetQ	protection	Tetracycline
117	tetW	protection	Tetracycline
118	tetX	deactivate	Tetracycline
119	tetS	protection	Tetracycline
120	tetbP	efflux	Tetracycline
121	tetR	tetR	Tetracycline
122	tetG_F	tetG_F	Tetracycline
123	tetPA	efflux	Tetracycline
124	tetM	protection	Tetracycline
125	tet44	tet44	Tetracycline
126	VanB	VanB	Vancomycin
127	vanD	protection	Vancomycin
128	vanHD	protection	Vancomycin
129	vanHB	protection	Vancomycin
130	vanRB	protection	Vancomycin
131	vanRD	protection	Vancomycin
132	vanTG	protection	Vancomycin
133	vanYD	protection	Vancomycin
