# Supplementary material for: Perinatal risk factors for fecal antibiotic resistance gene patterns in pregnant women and their infants
Source: PLoS One. 2020 Jun 18;15(6):e0234751. doi: 10.1371/journal.pone.0234751 (PMC7302573; doi:10.1371/journal.pone.0234751)
Supplement: S2 Table — (PDF) [file pone.0234751.s006.pdf]

Pregnancy samples	ARG			
**Race**	**White**		**Non-white**	**p-value**
Sum of abundance	0.56 (0.33 - 1.06)		2.62 (0.72 - 5.63)	0.062†
Richness	54 (42 - 60)		30.5 (29.3 - 49)	0.193
Shannon Index	1.8 (1.54 - 2.21)		1.36 (1.06 - 1.75)	0.099†
Inverse Simpson Index	4.39 (3.09 - 6.42)		2.78 (2.05 - 4.09)	0.149
**Parity**	**1-2 children**		**>3 children**	**p-value**
Sum of abundance	0.76 (0.40 - 1.55)		0.65 (0.46 - 1.39)	0.952
Richness	51.5 (32.5 - 58.5)		56.5 (53.5 - 61)	0.178
Shannon Index	1.71 (1.28 - 1.99)		1.96 (1.75 - 2.23)	0.198
Inverse Simpson Index	3.7 (2.7-5.4)		5.09 (3.68 - 6.91)	0.138
**Smoking**	**Never**		**Ever**	**p-value**
Sum of abundance	0.56 (0.29 - 1.47)		1.05 (0.51 - 1.58)	0.313
Richness	53 (34.5 - 59)		55 (36.8 - 62.5)	0.725
Shannon Index	1.76 (1.27 - 2.16)		1.79 (1.44 - 2.12)	0.53
Inverse Simpson Index	3.46 (2.41 - 5.66)		4.35 (3.2 - 6.48)	0.234
**Pre-Pregnancy BMI**	**Normal or underweight**	**Overweight**	**Obese**	**p-value**
Sum of abundance	0.83 (0.48 - 1.51)	0.56 (0.28 - 1.74)	0.57 (0.26 - 1.38)	0.613
Richness	55.5 (41.5 - 63.5)	53 (31 - 57)	52 (38 - 57.5)	0.659
Shannon Index	1.85 (1.59 - 2.43)	1.65 (1.27 - 2.35)	1.67 (1.43 - 1.9)	0.311
Inverse Simpson Index	4.77 (3.26 - 8.19)	3.46 (2.46 - 7.41)	3.52 (2.85 - 4.78)	0.293

Infancy samples	ARG		
**Sex**	**Female**	**Male**	**p-value**
Sum of abundance	0.95 (0.36 - 1.13)	0.51 (0.34 - 0.79)	0.252
Richness	48 (42 - 52)	51 (48 - 57.5)	0.056†
Shannon Index	2.21 (1.82 - 2.36)	2.17 (1.79 - 2.38)	0.887
Inverse Simpson Index	6.06 (3.47 - 7.74)	5.56 (3.9 - 8.48)	0.977
**Delivery mode**	**C-section**	**Vaginal**	**p-value**
Sum of abundance	0.47 (0.35 - 0.79)	0.58 (0.25 - 1.22)	0.332
Richness	53 (49.5 - 60)	49 (44 - 54)	0.059†
Shannon Index	2.17 (1.91 - 2.45)	2.2 (1.64 - 2.32)	0.761

Inverse Simpson Index	5.14 (4.07 - 8.4)	6.06 (3.47 - 7.94)	0.761
**Breastmilk percentage**	**<50%**	**≥50%**	**p-value**
Sum of abundance	0.51 (0.41 - 0.79)	0.59 (0.21 - 1.13)	0.719
Richness	49 (45 - 55.5)	51 (45 - 57)	0.812
Shannon Index	2.21 (1.84 - 2.39)	2.12 (1.82 - 2.36)	0.699
Inverse Simpson Index	6.06 (4.44 - 8.21)	5.97 (3.3 - 7.94)	0.741
**Diet**	**No solid food**	**Solid food**	**p-value**
Sum of abundance	0.56 (0.40 - 0.84)	0.52 (0.27 - 1.01)	0.635
Richness	58.5 (51.3 - 61)	50 (45 - 53)	0.107
Shannon Index	2.21 (1.73 - 2.47)	2.2 (1.76 - 2.35)	0.739
Inverse Simpson Index	5.92 (3.43 - 8.14)	6.19 (3.99 - 8.28)	0.902

† = p values < 0.1)
