# Supplementary material for: Perinatal risk factors for fecal antibiotic resistance gene patterns in pregnant women and their infants
Source: PLoS One. 2020 Jun 18;15(6):e0234751. doi: 10.1371/journal.pone.0234751 (PMC7302573; doi:10.1371/journal.pone.0234751)
Supplement: S3 Table — (PDF) [file pone.0234751.s007.pdf]

Pregnancy samples	MGE			
**Race**	**White**		**Non-white**	**p-value**
Sum of abundance	0.09 (0.03 - 0.28)		$4x10^{-4}$ ($3x10^{-5}$ - 0.03)	0.003*
Richness	11 (7 - 14)		4 (2.5 - 8.5)	0.035*
Shannon Index	0.64 (0.16 - 1.18)		0.87 (0.51 - 1.18)	0.909
Inverse Simpson Index	1.71 (1.06 - 2.77)		2.29 (1.38 - 3.05)	0.546
**Parity**	**1-2 children**		**>3 children**	**p-value**
Sum of abundance	0.06 (0.0026 - 0.21)		0.14 (0.09 - 0.25)	0.074†
Richness	9.5 (6 - 13)		11 (9.25 - 15)	0.185
Shannon Index	0.72 (0.28 - 1.24)		0.26 (0.10 - 1.09)	0.409
Inverse Simpson Index	1.73 (1.13 - 2.87)		1.14 (1.03 - 2.76)	0.382
**Smoking**	**Never**		**Ever**	**p-value**
Sum of abundance	0.07 (0.02 - 0.22)		0.11 (0.007 - 0.29)	0.557
Richness	11 (6.5 - 12.5)		10 (5.3 - 13)	0.936
Shannon Index	0.68 (0.16 - 1.23)		0.66 (0.20 - 1.15)	0.866
Inverse Simpson Index	1.73 (1.07 - 2.78)		1.6 (1.08 - 2.91)	0.788
**Pre-Pregnancy BMI**	**Normal or underweight**	**Overweight**	**Obese**	**p-value**
Sum of abundance	0.17 (0.05 - 0.32)	0.07 (0.001 - 0.15)	0.03 (0.004 - 0.15)	0.279
Richness	11.5 (7 - 13)	10 (4 - 14)	8 (5.5 - 12)	0.525
Shannon Index	0.63 (0.17 - 1.34)	0.75 (0.26 - 1.07)	0.71 (0.24 - 1.21)	0.967
Inverse Simpson Index	1.59 (1.06 - 3.03)	1.87 (1.13 - 2.71)	1.73 (1.11 - 2.82)	0.973

Infancy samples	MGE		
**Sex**	**Female**	**Male**	**p-value**
Sum of abundance	0.21 (0.12 - 0.79)	0.27 (0.07 - 0.52)	0.512
Richness	14 (13 - 15)	15 (12 - 16)	0.694
Shannon Index	1.23 (0.76 - 1.35)	1.32 (0.36 - 1.62)	0.819
Inverse Simpson Index	2.7 (1.94 - 3.04)	3.04 (1.17 - 3.96)	0.588
**Delivery mode**	**C-section**	**Vaginal**	**p-value**
Sum of abundance	0.36 (0.15 - 0.92)	0.20 (0.08 - 0.61)	0.361
Richness	15 (12.5 - 16)	14 (12 - 15)	0.439

Shannon Index	1.32 (0.35 - 1.75)	1.23 (0.75 - 1.47)	0.439
Inverse Simpson Index	3.04 (1.16 - 4.71)	2.73 (1.74 - 3.65)	0.439
**Breastmilk percentage**	**<50%**	**≥50%**	**p-value**
Sum of abundance	0.19 (0.09 - 0.44)	0.31 (0.08 - 0.79)	0.455
Richness	14 (12.5 - 16)	14 (12 - 15)	0.746
Shannon Index	1.21 (0.35 - 1.63)	1.24 (0.75 - 1.49)	0.912
Inverse Simpson Index	2.7 (1.15 - 3.95)	2.82 (1.74 - 3.67)	1
**Diet**	**No solid food**	**Solid food**	**p-value**
Sum of abundance	0.38 (0.27 - 0.54)	0.25 (0.08 - 0.79)	0.515
Richness	14.5 (13.5 - 15.3)	14 (13 - 16)	0.814
Shannon Index	1.26 (0.67 - 1.51)	1.28 (0.51 - 1.54)	0.986
Inverse Simpson Index	2.62 (1.76 - 3.89)	3.01 (1.38 - 3.75)	0.986

* = p values ≤ 0.05
† = p values (0.05 - 0.1)
