# Supplementary material for: Perinatal risk factors for fecal antibiotic resistance gene patterns in pregnant women and their infants
Source: PLoS One. 2020 Jun 18;15(6):e0234751. doi: 10.1371/journal.pone.0234751 (PMC7302573; doi:10.1371/journal.pone.0234751)
Supplement: S4 Table — Infancy samples (n = 41). (PDF) [file pone.0234751.s008.pdf]

Name	OTU	Pearson correlation
blaTEM	Fusobacteria	0.99
ISCR1	Lentisphaerae	0.97
aac(3)-Xa	Fusobacteria	0.98
IS26	Fusobacteria	0.99
sugE	Fusobacteria	0.98
Tn3	Fusobacteria	1.00
IncI1_repI1	Fusobacteria	1.00
IS91	Tenericutes	1.00
tetB	Fusobacteria	0.99
tnpA	Fusobacteria	0.95
tnpA	Fusobacteria	0.99
vanTG	Lentisphaerae	0.93
int1-a-marko	Fusobacteria	0.99
intI1F165_clinical	Fusobacteria	0.97
sul1 NEW	Fusobacteria	0.99
orf39-IS26	Synergistetes	0.93
aph6ic	Synergistetes	0.91
aadA17	Fusobacteria	0.99
tetR	Fusobacteria	0.99
dfra21	Fusobacteria	0.99
erm(E)	Tenericutes	0.99
