# Supplementary material for: Perinatal risk factors for fecal antibiotic resistance gene patterns in pregnant women and their infants
Source: PLoS One. 2020 Jun 18;15(6):e0234751. doi: 10.1371/journal.pone.0234751 (PMC7302573; doi:10.1371/journal.pone.0234751)
Supplement: S5 Table — Pregnancy samples (n = 51). (PDF) [file pone.0234751.s009.pdf]

Gene	OTU	Pearson correlation
blaOXY-1	Lentisphaerae	0.87
mefA	Lentisphaerae	0.89
blaTEM	Fusobacteria	0.99
aac(3)-Xa	Fusobacteria	0.98
IS26	Fusobacteria	0.99
sugE	Fusobacteria	0.98
tetA	Fusobacteria	0.88
tnpA	Fusobacteria	0.95
oprD	Lentisphaerae	0.87
sulA/folP	Synergistetes	0.86
intI1F165_clinical	Fusobacteria	0.97
aph6ic	Synergistetes	0.91
erm(E)	Tenericutes	0.99
mphA	Fusobacteria	0.87
