# Supplementary material for: Perinatal risk factors for fecal antibiotic resistance gene patterns in pregnant women and their infants
Source: PLoS One. 2020 Jun 18;15(6):e0234751. doi: 10.1371/journal.pone.0234751 (PMC7302573; doi:10.1371/journal.pone.0234751)
Supplement: S6 Table — Infancy samples (n = 41). (PDF) [file pone.0234751.s010.pdf]

Gene	OTU	Pearson correlation
blaCTX-M-1,3,15	Neisseriaceae	0.83
blaCTX-M-1,3,15	Leptotrichiaceae	0.99
blaTEM	Leuconostocaceae	0.83
tetM	Planococcaceae	0.89
tetM	Bacilli_unclassified	0.88
bla-ACT	Caulobacteraceae	0.99
bla-ACT	Flavobacteriaceae	0.99
bla-ACT	Acidaminococcaceae	0.85
ermX	Actinobacteria_unclassified	0.87
ermX	Thermoanaerobacteraceae	0.87
ermX	Opitutae_unclassified	0.87
oprD	Gammaproteobacteria_unclassified	1.00
oprD	Moraxellaceae	0.84
oprD	Comamonadaceae	1.00
pmrA	Lactobacillales_unclassified	0.81
ermA/ermTR	Rhodospirillaceae	1.00
intI1F165_clinical	Gammaproteobacteria_unclassified	0.84
intI1F165_clinical	Comamonadaceae	0.84
aac(3)-iid_iii_iif_iia_iie	Gastranaerophilales_fa	1.00
aac(3)-iid_iii_iif_iia_iie	Rikenellaceae	0.82
ere(A)	Staphylococcaceae	0.81
ere(A)	Eubacteriaceae	0.86
