# Supplementary material for: Perinatal risk factors for fecal antibiotic resistance gene patterns in pregnant women and their infants
Source: PLoS One. 2020 Jun 18;15(6):e0234751. doi: 10.1371/journal.pone.0234751 (PMC7302573; doi:10.1371/journal.pone.0234751)
Supplement: S7 Table — Pregnancy samples (n = 51). (PDF) [file pone.0234751.s011.pdf]

Gene	OTU	Pearson correlation
blaOXY-1	Victivallaceae	0.87
aph6ia	Puniceicoccaceae	0.95
mefA	Victivallaceae	0.90
blaTEM	Corynebacteriaceae	0.85
blaTEM	Fusobacteriaceae	0.99
czcA	Pseudomonadaceae	0.80
aph3-III	unclassified.Burkholderiales	0.84
aac(3)-Xa	Corynebacteriaceae	0.83
aac(3)-Xa	Fusobacteriaceae	0.98
IS26	Corynebacteriaceae	0.85
IS26	Fusobacteriaceae	0.99
sugE	Corynebacteriaceae	0.84
sugE	Fusobacteriaceae	0.98
IS1247	Staphylococcaceae	0.82
tetA	Fusobacteriaceae	0.88
tnpA	Corynebacteriaceae	0.82
tnpA	Fusobacteriaceae	0.95
ermX	Pasteurellaceae	0.81
ermX	Streptococcaceae	0.82
vanHD	Staphylococcaceae	0.85
oprD	Victivallaceae	0.88
mexE	Puniceicoccaceae	0.99
sulA/folP	Synergistaceae	0.86
intI1F165_clinical	Corynebacteriaceae	0.82
intI1F165_clinical	Fusobacteriaceae	0.97
ISEfm1-Entero	Enterococcaceae	0.82
aph6ic	Synergistaceae	0.91
aadA7	Bacteroidales_S24.7_group	0.96
tet(32)	unclassified.Burkholderiales	0.84
erm(E)	Mollicutes_RF9_fa	0.99
erm(E)	Clostridiales_vadinBB60_group	0.81
mphA	Fusobacteriaceae	0.87
