# Supplementary material for: Perinatal risk factors for fecal antibiotic resistance gene patterns in pregnant women and their infants
Source: PLoS One. 2020 Jun 18;15(6):e0234751. doi: 10.1371/journal.pone.0234751 (PMC7302573; doi:10.1371/journal.pone.0234751)
Supplement: S8 Table — Infancy samples (n = 41). (PDF) [file pone.0234751.s012.pdf]

Gene	OTU	Pearson correlation
cfxA	Tyzzerella	0.90
acrF	Escherichia/Shigella	0.83
blaCTX-M-1,3,15	Ruminococcaceae_UCG.014	0.99
blaCTX-M-1,3,15	Butyricicoccus	0.83
blaCTX-M-1,3,15	Leptotrichia	0.99
blaCTX-M-1,3,15	Neisseria	0.85
blaCTX-M-1,3,15	uncultured	0.99
blaOXY-1	Anaeroglobus	0.92
blaOXY-1	Cellulosilyticum	0.97
mdth	Escherichia/Shigella	0.83
pcoA	Atopobium	0.98
pcoA	Scardovia	0.98
blaTEM	Weissella	0.83
tetM	Rikenellaceae_RC9	0.89
tetM	Rummeliibacillus	0.89
bla-ACT	Desulfovibrio	0.98
bla-ACT	Candidatus_Soleaferrea	0.99
bla-ACT	Neorhizobium	0.99
bla-ACT	Lactococcus	0.87
bla-ACT	Brevundimonas	0.99
bla-ACT	Phascolarctobacterium	0.91
bla-ACT	Empedobacter	0.99
IS26	Scardovia	0.91
IS26	Atopobium	0.91
sugE	Dielma	0.85
sugE	Ruminococcaceae	0.88
sugE	Fusicatenibacter	0.82
IS200_1	Dielma	0.97
IS200_1	Ruminococcaceae	0.97
IS200_1	Fusicatenibacter	0.96
IS200	Hungatella	0.88
IS200	Providencia	1.00
oqxA	Atopobium	0.96
oqxA	Scardovia	0.95

ermX	Ruminococcaceae_UCG.003	0.87
ermX	Gelria	0.87
ermX	Ruminococcaceae_UCG.002	0.83
oprD	Acinetobacter	0.84
ermA/ermTR	Dialister	0.80
tolC	Escherichia/Shigella	0.84
merA-marko	Atopobium	0.99
merA-marko	Scardovia	0.99
orf39-IS26	Atopobium	0.97
orf39-IS26	Scardovia	0.97
aac(3)-iid_iii_iif_iia_iie	Gastranaerophilales_ge	1.00
aac(3)-iid_iii_iif_iia_iie	Actinotignum	1.00
aac(3)-iid_iii_iif_iia_iie	Varibaculum	0.91
aac(3)-iid_iii_iif_iia_iie	Peptoniphilus	0.98
aac(3)-iid_iii_iif_iia_iie	Negativicoccus	0.92
aac(3)-iid_iii_iif_iia_iie	Alistipes	0.82
aac(3)-iid_iii_iif_iia_iie	Peptostreptococcus	1.00
emrD	Cellulosilyticum	0.91
emrD	Anaeroglobus	0.85
ere(A)	Ruminiclostridium_9	0.92
ere(A)	Staphylococcus	0.81
ere(A)	Eubacterium	0.85
mdtE/yhiU	Escherichia/Shigella	0.86
acrB	Escherichia/Shigella	0.87
