# Supplementary material for: Perinatal risk factors for fecal antibiotic resistance gene patterns in pregnant women and their infants
Source: PLoS One. 2020 Jun 18;15(6):e0234751. doi: 10.1371/journal.pone.0234751 (PMC7302573; doi:10.1371/journal.pone.0234751)
Supplement: S9 Table — Pregnancy samples (n = 51). (PDF) [file pone.0234751.s013.pdf]

Gene	OTU	Pearson correlation
blaOXY-1	Victivallis	0.87
blaOXY-1	Alloprevotella	0.92
blaMIR	Coprococcus_2	0.82
aph6ia	Pseudobutyrivibrio	0.95
mefA	Victivallis	0.89
mefA	Alloprevotella	0.96
blaTEM	Mobiluncus	0.99
blaTEM	Lawsonella	0.89
blaTEM	Moryella	0.99
blaTEM	Porphyromonas	0.94
blaTEM	Fastidiosipila	0.99
blaTEM	Fusobacterium	0.99
blaTEM	Ezakiella	0.97
blaTEM	Corynebacterium_1	0.99
blaTEM	Prevotella_6	0.99
blaTEM	Hungatella	0.90
blaTEM	Tyzzerella_4	0.98
blaTEM	Anaerococcus	0.88
blaTEM	Parvimonas	0.99
blaTEM	uncultured.9	0.99
blaTEM	Finegoldia	0.99
blaTEM	Peptoniphilus	0.99
blaTEM	Jonquetella	0.99
blaTEM	Prevotella	0.91
blaTEM	Murdochiella	0.93
intl3	Chroococcidiopsis	0.83
czcA	Chroococcidiopsis	0.94
czcA	Pseudomonas	0.80
aac(3)-Xa	Tyzzerella_4	0.96
aac(3)-Xa	Fastidiosipila	0.98
aac(3)-Xa	Fusobacterium	0.98
aac(3)-Xa	Anaerococcus	0.87
aac(3)-Xa	Mobiluncus	0.98
aac(3)-Xa	Parvimonas	0.98

aac(3)-Xa	Jonquetella	0.98
aac(3)-Xa	Ezakiella	0.95
aac(3)-Xa	Lawsonella	0.88
aac(3)-Xa	Peptoniphilus	0.97
aac(3)-Xa	Finegoldia	0.98
aac(3)-Xa	Moryella	0.98
aac(3)-Xa	Murdochiella	0.92
aac(3)-Xa	Hungatella	0.91
aac(3)-Xa	Porphyromonas	0.92
aac(3)-Xa	uncultured.9	0.98
aac(3)-Xa	Prevotella	0.94
aac(3)-Xa	Corynebacterium_1	0.98
aac(3)-Xa	Prevotella_6	0.98
IS26	Anaerococcus	0.88
IS26	Prevotella_6	0.99
IS26	Fastidiosipila	0.99
IS26	Parvimonas	0.99
IS26	Fusobacterium	0.99
IS26	Murdochiella	0.95
IS26	Tyzzerella_4	0.99
IS26	Moryella	0.99
IS26	Corynebacterium_1	0.99
IS26	Ezakiella	0.97
IS26	Prevotella	0.93
IS26	Peptoniphilus	0.99
IS26	Porphyromonas	0.94
IS26	Jonquetella	0.99
IS26	Mobiluncus	0.99
IS26	Hungatella	0.94
IS26	Finegoldia	0.99
IS26	Lawsonella	0.89
IS26	uncultured.9	0.99
sugE	Fastidiosipila	0.98
sugE	Moryella	0.98
sugE	Anaerococcus	0.88
sugE	Tyzzerella_4	0.97
sugE	Jonquetella	0.98
sugE	Mobiluncus	0.98
sugE	Finegoldia	0.98

sugE	Fusobacterium	0.98
sugE	Parvimonas	0.98
sugE	Murdochiella	0.92
sugE	Prevotella	0.90
sugE	Peptoniphilus	0.98
sugE	Ezakiella	0.96
sugE	Prevotella_6	0.98
sugE	Hungatella	0.89
sugE	Porphyromonas	0.93
sugE	Lawsonella	0.89
sugE	Corynebacterium_1	0.98
IS1247	Staphylococcus	0.81
IS1247	Faecalicoccus	0.95
IS1247	Mogibacterium	0.87
aadE	Ruminococcaceae_UCG.011	0.84
tetA	Finegoldia	0.88
tetA	Jonquetella	0.88
tetA	Prevotella	0.81
tetA	Murdochiella	0.82
tetA	Tyzzerella_4	0.87
tetA	Fusobacterium	0.88
tetA	Fastidiosipila	0.88
tetA	Corynebacterium_1	0.88
tetA	Prevotella_6	0.88
tetA	Mobiluncus	0.88
tetA	Porphyromonas	0.84
tetA	Parvimonas	0.88
tetA	Ezakiella	0.88
tetA	Peptoniphilus	0.88
tetA	Moryella	0.88
tnpA	Corynebacterium_1	0.95
tnpA	Moryella	0.95
tnpA	Jonquetella	0.95
tnpA	Fusobacterium	0.95
tnpA	Porphyromonas	0.91
tnpA	Prevotella_6	0.94
tnpA	Anaerococcus	0.84
tnpA	Tyzzerella_4	0.95
tnpA	Peptoniphilus	0.94

tnpA	Ezakiella	0.93
tnpA	Mobiluncus	0.95
tnpA	Prevotella	0.91
tnpA	Finegoldia	0.95
tnpA	Parvimonas	0.95
tnpA	Fastidiosipila	0.95
tnpA	Hungatella	0.94
tnpA	Murdochiella	0.94
tnpA	Lawsonella	0.85
ermX	Klebsiella	0.98
ermX	Paeniclostridium	1.00
ermX	Haemophilus	0.82
ermX	Alloscardovia	0.81
ermX	Lactococcus	0.99
ermX	Aggregatibacter	1.00
vanHD	Faecalicoccus	0.98
vanHD	Staphylococcus	0.84
vanHD	Mogibacterium	0.89
oprD	Victivallis	0.87
oprD	Alloprevotella	0.93
oprD	uncultured_ge	0.91
mexE	Pseudobutyrivibrio	0.99
sulA/folP	Dysgonomonas	0.90
sulA/folP	Cloacibacillus	0.81
sulA/folP	Pyramidobacter	0.90
ermA/ermTR	Coprococcus_2	0.82
intI1F165_clinical	Peptoniphilus	0.96
intI1F165_clinical	Fastidiosipila	0.97
intI1F165_clinical	Tyzzerella_4	0.95
intI1F165_clinical	Jonquetella	0.97
intI1F165_clinical	Fusobacterium	0.97
intI1F165_clinical	Prevotella	0.88
intI1F165_clinical	Corynebacterium_1	0.97
intI1F165_clinical	Hungatella	0.90
intI1F165_clinical	Anaerococcus	0.86
intI1F165_clinical	Ezakiella	0.94
intI1F165_clinical	Mobiluncus	0.97
intI1F165_clinical	Porphyromonas	0.91
intI1F165_clinical	Parvimonas	0.97

intI1F165_clinical	Murdochiella	0.91
intI1F165_clinical	Moryella	0.97
intI1F165_clinical	Finegoldia	0.97
intI1F165_clinical	Lawsonella	0.87
intI1F165_clinical	Prevotella_6	0.96
ISEfm1-Entero	Enterococcus	0.85
aph6ic	Cloacibacillus	0.86
aph6ic	Ruminiclostridium_1	0.87
aph6ic	Dysgonomonas	0.96
aph6ic	Pyramidobacter	0.96
aac(6)-im	Olsenella	0.86
aac(6)-im	Mitsuokella	0.93
aadA7	Bacteroidales_S24.7	0.96
aadA7	Ruminococcaceae_UCG.008	0.86
tetG_F	Faecalicoccus	0.85
erm(E)	Peptococcus	0.98
erm(E)	Enterorhabdus	1.00
erm(E)	NB1.n_ge	0.98
erm(E)	Anaerotruncus	0.95
erm(E)	Mollicutes_RF9_ge	0.99
erm(E)	Clostridiales_vadinBB60	0.81
erm(E)	Holdemanella	0.84
mphA	Prevotella	0.93
mphA	Prevotella_6	0.89
mphA	Moryella	0.87
mphA	Fusobacterium	0.87
mphA	Tyzzerella_4	0.85
mphA	Porphyromonas	0.82
mphA	Jonquetella	0.87
mphA	Murdochiella	0.80
mphA	Corynebacterium_1	0.87
mphA	Finegoldia	0.87
mphA	Mobiluncus	0.87
mphA	Ezakiella	0.84
mphA	Parvimonas	0.87
mphA	Fastidiosipila	0.87
mphA	Peptoniphilus	0.86
